# NHC-Au-Xanthate Complexes: Selective Cytotoxic Activity and Mechanistic Insights

**DOI:** 10.1021/acsomega.6c07778

**Published:** 2026-10-12

**Authors:** Katarzyna Niemirowicz-Laskowska, Marta Pietruszyńska, Supratim Chakraborty, Piotr F. J. Lipiński, Wioletta Kośnik, Beata Skonieczna, Iwona Misztalewska-Turkowicz, Halina Car, Michał Michalak

**Affiliations:** † Department of Experimental Pharmacology, Medical University of Białystok, Szpitalna 37, 15-295 Bialystok, Poland; ‡ Faculty of Chemistry, Medical University of Bialystok, Waszyngtona 15A, 15-245 Bialystok, Poland; § Institute of Organic Chemistry, Polish Academy of Sciences, Kasprzaka 44/52, 01-224 Warsaw, Poland; ∥ Department of Neuropeptides, Mossakowski Medical Research Institute, Polish Academy of Sciences, Pawinskiego 5, 02-106 Warsaw, Poland; ⊥ NanoVelos S.A., Rakowiecka 36, 02-532 Warsaw, Poland; # Faculty of Chemistry, University of Bialystok, Ciolkowskiego 1K, 15-274 Bialystok, Poland

## Abstract

In this study, a series of NHC-Au-xanthate (NHC-Au-X_a_) complexes was evaluated for their antiproliferative activity and mechanism of action. Molecular docking studies identified thioredoxin reductase (TrxR) as a plausible molecular target and suggested that the inhibitory potency may depend on the ability of the complexes to position the gold center in close proximity to the catalytic selenocysteine residue. Consistent with this hypothesis, the investigated compounds induced TrxR inhibition, leading to an intracellular redox imbalance, enhanced reactive oxygen species (ROS) generation, increased release of damage-associated molecular patterns (DAMPs), including HMGB1, and activation of both apoptotic and necrotic cell death pathways. The aforementioned mechanism might indicate the engagement of immunologically relevant cell death responses. Additionally, fluorescence studies demonstrated the preferential accumulation of a representative complex in cancer cells compared with normal fibroblasts. In conclusion, these findings provide new insights into the mechanism of action of NHC-Au-xanthate complexes and highlight their potential as redox-active anticancer agents with possible chemoimmunotherapeutic relevance.

## Graphical abstract

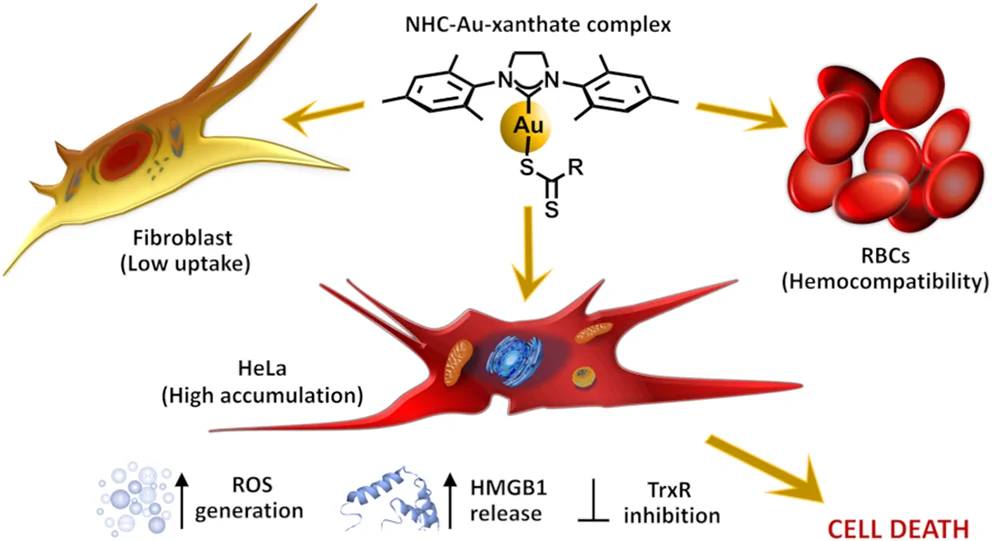

## Introduction

Metallodrugs currently constitute an emerging alternative to small organic molecules in cancer treatment, mainly due to their complementary multimodal mechanisms of action.^1,2^ This has led to the successful development of platinum-based therapies that suppress DNA replication and transcription. Unfortunately, the widespread use of platinum metallodrugs has also resulted in increased platinum resistance in some cancer cell lines.^3,4^ Thus, there is a growing need for new metallodrugs based on alternative mechanisms of action. Among metal complexes, gold compounds appear to be the most promising, as they can strongly bind to selenoenzymes such as thioredoxin reductase (TrxR),^5–10^ which exists in the cytosol and mitochondria and is usually overexpressed in tumor cells. In particular, the cysteine residue located near a selenocysteine in TrxR enables organometallic complexes to bind to thiol and selenol groups, directly influencing the regulation of reactive oxygen species (ROS) and thereby inducing apoptosis. Among the wide variety of gold complexes bearing structurally and electronically diverse ligands,^11^ *N*-heterocyclic carbene (NHC) ligands secure an excellent balance between targeted lipophilicity, metabolic stability under physiological conditions due to the strong C_carbene_–Au bond, as well as ease of preparation and structural diversification. Although numerous studies have focused on structural modifications of NHC ligands of NHC-Au-Cl complexes in view of biological activity,^12–15^ further progress could be achieved by incorporating ligands that enable rapid binding of NHC-Au species to TrxR through the release of ancillary ligands (Figure 1).^16–22^

**Figure 1:**
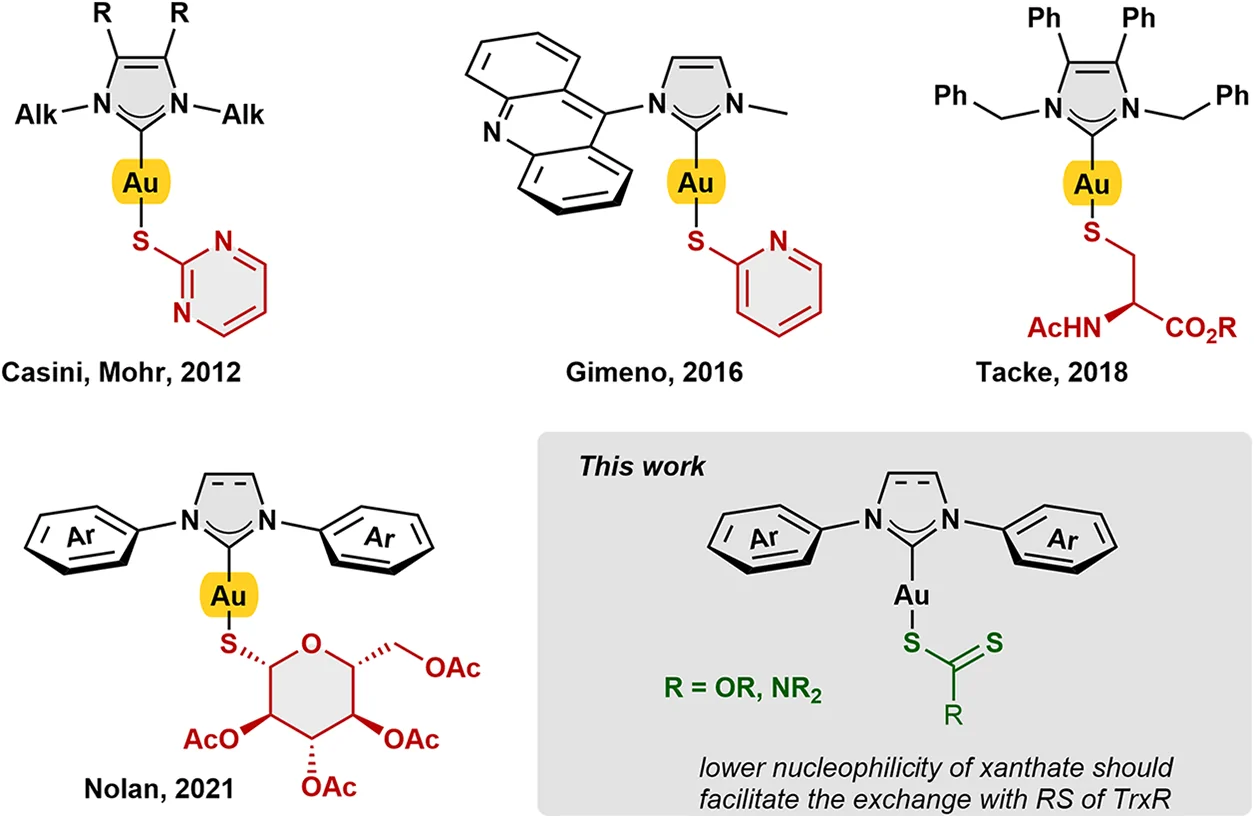
Selected examples of NHC-Au-thiolate complexes with proven biological activity.

Although NHC-Au­(I) complexes bearing chloride,^14^ alkynyl, and closely related ligands such as thiolate^23–28^ and selenoolate^29^ ligands, are well represented in medicinal chemistry, NHC-Au-xanthate complexes remain comparatively unexplored in biological applications. We recently reported a series of NHC-Au-xanthate (NHC-Au-X_a_) complexes (Figure 2)^30^ with primary focus on their applications in photocatalytic and π-catalyzed transformations. These studies demonstrated their synthetic accessibility, chemical stability, and high catalytic activity; however, their biological properties were not investigated. In addition to their stability and modular structure, an important potential advantage of these complexes over conventional NHC-Au-(seleno)­thiolate derivatives is their anticipated susceptibility to ligand exchange with biologically relevant sulfur- and selenium-containing nucleophiles.

**Figure 2:**
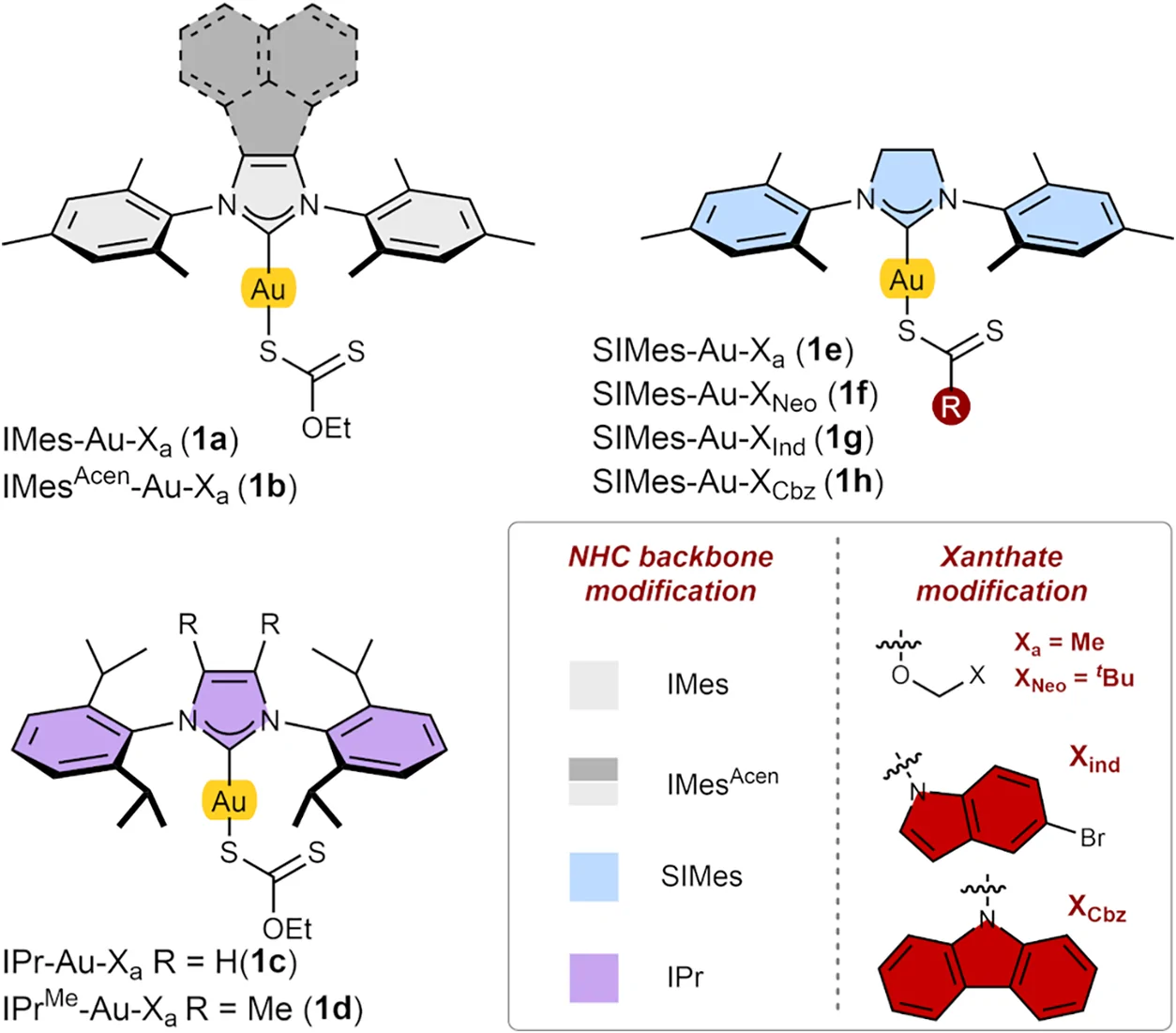
Structure of the investigated NHC-Au-xanthate complexes.

Our design rationale was based on the substantial difference in nucleophilicity between xanthate and thiolate species. According to Mayr’s nucleophilicity scale, the nucleophilicity parameter N is approximately 18–19 for xanthate anions and approximately 23 for thiophenolate anions.^31^ Consequently, the greater nucleophilicity of thiolate and, particularly, selenolate species is therefore expected to favor substitution of the xanthate ligand at the Au­(I) center. In contrast to conventional NHC-Au-thiolate complexes, in which the ancillary thiolate may bind more persistently to Au­(I), the xanthate ligand may provide a favorable balance between complex stability and susceptibility to ligand exchange. This property could facilitate the transfer of the NHC-Au­(I) fragment to the Cys/Sec-containing active site of thioredoxin reductase (TrxR). Given the frequent overexpression of TrxR in malignant cells and its central role in maintaining cellular redox homeostasis, we hypothesized that NHC-Au-X_a_ complexes could display pronounced anticancer activity and selectivity toward cancer cells. The present study, therefore, provides the first systematic biological and mechanism-oriented evaluation of this modular NHC-Au-xanthate series.

To further define the biological potential of NHC-Au-X (X = xanthate) complexes, we report here a comprehensive study of their cytotoxicity and the mechanisms underlying their cellular activity. HeLa cells, a well-established and extensively characterized cancer model widely used for the preliminary evaluation of novel anticancer compounds, together with fibroblasts and erythrocytes, were selected as complementary model systems to assess the antiproliferative activity, cytotoxicity, and potential mechanisms of action of the investigated complexes.^32^ The observed intracellular ROS generation, HMGB1 release, and induction of both apoptotic and necrotic cell death may indicate the involvement of immunogenic cell death (ICD)-related pathways. Indeed, gold complexes have recently emerged as promising ICD inducers due to their ability to perturb cellular redox homeostasis and activate stress responses associated with immune stimulation.^33,34^ In this context, the biological effects observed for the investigated complexes may extend beyond direct cytotoxicity and suggest a potential capacity to stimulate antitumor immune responses.

## Results and Discussion

### Effects on Cellular Viability and Proliferation

With the aim of exploring the potential anticancer effects of the NHC-Au-xanthates, we subjected a selection of human normal cell fibroblasts, CCD-1079Sk, and cervical cancer cell lines HeLa to a treatment with the synthesized compounds for different time periods, applied at concentrations ranging from 0.1 to 10 μM. Subsequently, cellular viability was assessed using the neutral red uptake (NRU) assay, a well-established method based on the selective accumulation of neutral red in the lysosomal compartments of metabolically active cells (Figure 3A,B).^35^ In all cases, a pronounced drop in the viability of cancer cells was observed, with submicromolar IC_10_ concentrations (as low as around 0.1 μM). In contrast, normal cells were not affected to such an extent even in concentrations up to 10 μM. In a subsequent step, proliferation assays were used to evaluate the effects on cell growth and division (Table 1). Consistent with the cytotoxic (and not cytostatic) effect, the tested NHC-Au-X_a_ complexes, in addition to affecting viability, significantly reduced proliferation.

**Figure 3:**
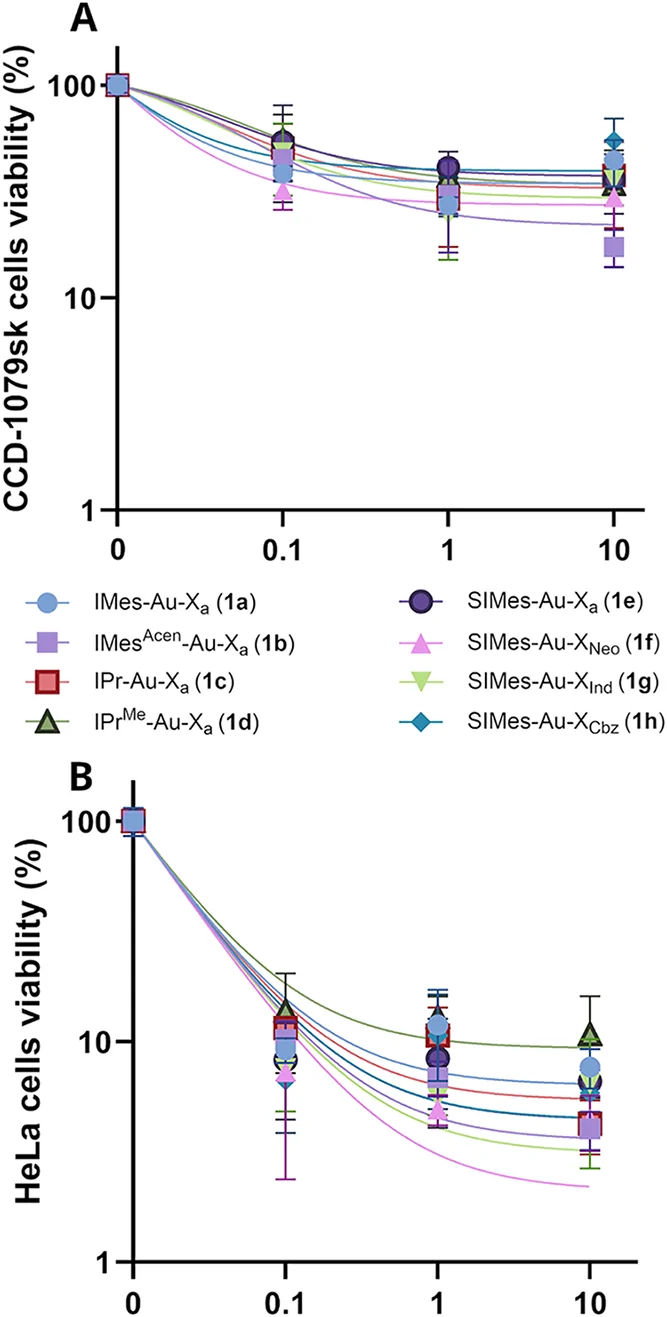
Effect of the test compounds on the viability of human fibroblast cells (A) and human cervical cancer cells (B) after 48 h exposure (error bars represent the standard deviation).

**Table 1:** Effect of the Tested Compounds on Cell Proliferation^a^

complexes	IC_50_ _HeLa_	IC_50_ _CCD‑1079Sk_	selectivity indexes (SI)
IMes-Au-X_a_ (**1a**)	0.24 ± 0.27	2.67 ± 0.77	11.12
IMes^Acen^-Au-X_a_ (**1b**)	0.07 ± 0.04	4.97 ± 3.2	71
IPr-Au-X_a_ (**1c**)	0.74 ± 0.27	1.98 ± 0.54	2.68
IPr^Me^-Au-X_a_ (**1d**)	0.93 ± 0.15	1.50 ± 0.34	1.61
SIMes-Au-X_a_ (**1e**)	0.76 ± 0.06	1.55 ± 0.045	2.03
SIMes-Au-X_Neo_ (**1f**)	0.12 ± 0.04	3.02 ± 0.11	25.15
SIMes-Au-X_Ind_ (**1g**)	57.44 ± 1.10	49.27 ± 7.38	0.85
SIMes-Au-X_Cbz_ (**1h**)	2.37 ± 2.6	25.12 ± 0.51	10.5

a IC_50_ is the half-maximal inhibitory concentration [μM]. The antiproliferative activity of the investigated complexes was evaluated in HeLa cervical cancer cells and normal human fibroblasts CCD-1079Sk following 48 h exposure. IC_50_ values were determined by fitting concentration–response curves using a nonlinear regression model.

The complexes exhibit significant toxicity toward cancer cells (IC_50_ in the range 0.07–2.37 μM). Based on the obtained results, it can be concluded that the tested complexes exhibited higher selectivity toward cervical cancer cells than human fibroblasts (SI index >1). Complex **1b** is the most potent (IC_50_ HeLa = 0.07 μM) and at the same time the most selective (71-fold difference in IC_50_ values). Notably, in addition to complex **1b**, complexes **1f** and **1a** demonstrated submicromolar antiproliferative activity (IC_50_ < 1 μM) accompanied by a favorable selectivity profile against representatives of normal fibroblast cells. Collectively, these results demonstrate that several of the investigated complexes combine remarkable antiproliferative potency with favorable cancer cell selectivity, warranting further mechanistic studies to elucidate the molecular pathways underlying their anticancer activity.

### Hemocompatibility

The potential for subsequent successful clinical application of these agents is dependent, to a considerable extent, on their hemocompatibility. In order to assess this important parameter, a hemolysis assay was conducted to evaluate the capacity of the NHC-Au-X_a_ complexes to disrupt the plasma membrane of red blood cells. The results clearly indicated (Table 2) that all agents exhibited compatibility with red blood cells (hemolysis ranging from 0.5% to 4%) when applied at a concentration as high as 100 μM, which is significantly higher than the IC_50_ values obtained for HeLa cells (submicromolar to low micromolar values). Overall, this suggests that the tested complexes are devoid of this serious liability. Notably, complex **1f** exhibited the lowest hemolytic activity among the investigated compounds, inducing only 0.5% hemolysis following 1 h incubation with human red blood cells. This finding is consistent with its previously demonstrated selectivity toward normal cells and further supports its favorable biocompatibility profile.

**Table 2:** Hemocompatibility of the Tested Compounds^a^

complexes	hemolysis (%)
IMes-Au-X_a_ (**1a**)	1.44 ± 0.98
IMesAcen-Au-X_a_ (**1b**)	1.32 ± 0.77
IPr-Au-X_a_ (**1c**)	1.89 ± 1.60
IPrMe-Au-X_a_ (**1d**)	1.54 ± 0.53
SIMes-Au-X_a_ (**1e**)	1.85 ± 0.16
SIMes-Au-X_Neo_ (**1f**)	0.54 ± 0.51
SIMes-Au-X_Ind_ (**1g**)	2.23 ± 0.02
SIMes-Au-X_Cbz_ (**1h**)	4.15 ± 0.06

a The hemolytic activity of the investigated NHC-Au-xanthate complexes was evaluated using fresh human erythrocytes. The results are the average ± standard deviation.

### *K*_i_-67 Levels

*K*_i_-67 expression has been identified as a reliable indicator of cell division rate and a prognostic factor that can effectively predict patient response to treatment and survival outcomes.^36^ In order to verify the capacity of the agents under investigation to inhibit the proliferation of HeLa cells, the expression levels of the *K*_i_-67 marker were determined. Auranofin was utilized as a positive control. As illustrated in Figure 4A, all but one (IMes^Acen^-Au-X_a_ **1b**) of the examined compounds restricted the proliferation of cells at concentrations similar to their IC_50_. It is particularly noteworthy that complexes **1f**, **1h**, **1c**, and **1d** exhibited a significant suppression of *K*_i_-67 expression, reaching levels comparable to those achieved by auranofin, applied at a concentration of 1 μM, which was chosen on the basis of literature reports demonstrating its antiproliferative activity in HeLa cells under comparable experimental conditions.^37^ In light of the previously documented potent cytotoxic activity and favorable selectivity profile of the compounds under study, the results further support their capacity to effectively impair the proliferative potential of cervical cancer cells. Since *K*_i_-67 is an indicator of cell-cycle activity rather than a direct measure of population doubling, these results should be interpreted as evidence of reduced proliferative status.

**Figure 4:**
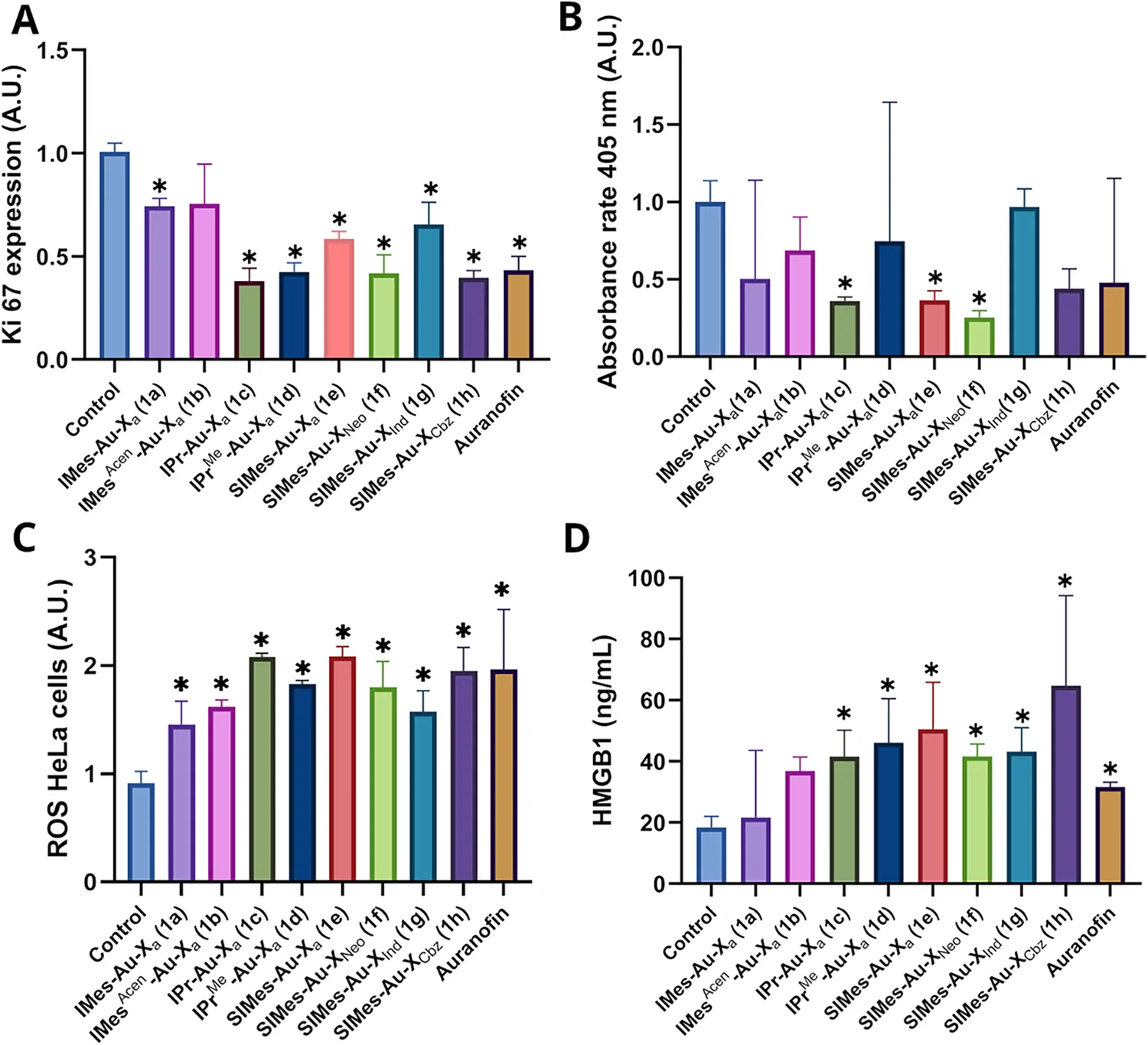
(A) The effect of the test compounds and auranofin [1 μM] on Ki67 expression after 48 h treatment of HeLa cells at their IC_50_ concentrations. (B) The effect of the test compounds on TrxR inhibition, expressed as a decrease in the absorbance rate at 405 nm, induced by treatment of the TrxR protein with 1 μM of the tested agents. (C) The effect of the test compounds on ROS production in HeLa cells. Fold change in ROS generation by HeLa cells after 24 h treatment with complexes at a concentration of 0.5 μM. ROS levels were determined using the ROS-Glo H_2_O_2_ Assay (Promega). (D) extracellular HMGB1 concentration after 24 h treatment with the complexes at a concentration of 1 μM. HMGB1 levels were determined using the Lumit HMGB1 Immunoassay (Promega). Statistical significance: vs control was marked (*) *p* ≤ 0.05. The data presented constitute average results from three measurements ± SD. Error bars represent the standard deviation.

### Mechanistic Insights: TrxR Inhibition, Effect on Reactive Oxygen Species Levels and HMGB1 Levels

In recent decades, growing evidence has emerged regarding the preclinical mechanistic effects of auranofin on cancer cells.^38^ The hypothesis proposes that the primary mechanism of action is its ability to inhibit thioredoxin reductase (TrxR),^39^ which is represented by cytoplasmic and mitochondrial isoforms. It should be emphasized that TrxR is frequently overexpressed in cancerous cells compared to normal cells. In effect, this enzyme is an emerging pharmacologic target in cancer treatment.^40^

Hence, our complexes were tested for the inhibition of TrxR (Figure 4B). At 1 μM, SIMes-Au-X_Neo_ (**1f**), IMes-Au-X_a_ (**1a**), and IPr-Au-X_a_ (**1c**) significantly inhibited the enzyme activity. These effects were at a level similar to that shown by auranofin. Compound SIMes-Au-X_Neo_ (**1f**) was the most potent inhibitor. On the contrary, SIMes-Au-X_Ind_ (**1g**) exhibited no sign of inhibitory activity. Inhibition of thioredoxin reductase disrupts the thioredoxin-dependent antioxidant system, preventing efficient regeneration of reduced thioredoxin and impairing peroxide detoxification by peroxiredoxins. Hence, we investigated the ability of the tested agents to induce ROS production in treated cancer cells via a luminescence-based method. Pleasingly, all compounds exhibited a 1.5- to 2-fold increase in ROS production in treated cells compared to the control (Figure 4C).

Importantly, our results indicated that in different complexes, the TrxR inhibition is not necessarily correlated with the effect on ROS. Complexes **1f**, **1e**, and **1c** both increase ROS production and inhibit TrxR. On the contrary, other derivatives elevated the ROS level without significantly affecting the expected target enzyme. The above-mentioned findings could suggest that an alternative mode of action might operate. Depending on the severity and duration of the oxidative stress, as well as other stimuli, different cancer cell death pathways can be modulated.^41^

Given the well-established susceptibility of TrxR to inhibition by gold compounds,^33,34^ the observed biological effects may originate from the disruption of the thioredoxin antioxidant system. It is evident that the Trx/TrxR system plays a pivotal role in the regulation of a multitude of redox-sensitive cellular proteins. The system has been demonstrated to modulate signaling cascades and impact a variety of cellular processes, including proliferation, differentiation, and apoptosis.^42^ Consequently, its inhibition has the potential to compromise antioxidant defense mechanisms, leading to an accumulation of ROS. To investigate whether the observed oxidative stress was associated with downstream danger signaling, HMGB1 levels were also determined. HMGB1 is a redox-sensitive nonhistone nuclear protein that can translocate from the nucleus and be released extracellularly under conditions of oxidative stress and cell death.^43^ HMGB1 contains three cysteine residues (C23, C45, and C106) and can exist in distinct redox isoforms, including reduced, disulfide, oxidized, and dimerized forms. The redox state of HMGB1 regulates its localization and biological functions, including its involvement in DNA damage responses, inflammation, cell death, and cell survival.^44^ In agreement with the proposed mechanism, treatment with the investigated complexes resulted in an approximately 2-fold increase in ROS generation accompanied by a two- to 3-fold increase in HMGB1 secretion. Elevated extracellular HMGB1 levels were observed for all compounds except **1a** and **1b** (Figure 4D), supporting the involvement of damage-associated molecular pattern (DAMP) signaling pathways.^45^ Collectively, these findings support the conclusion that oxidative stress induced by the investigated gold complexes promotes HMGB1 release and DAMP signaling, thereby potentially contributing to the initiation of immunogenic cell death pathways.^46^ The observed HMGB1 release may be associated with the redox imbalance resulting from TrxR inhibition and an increased level of ROS generation. While the redox state of extracellular HMGB1 was not determined in the present study, the concomitant TrxR inhibition, ROS accumulation, and HMGB1 release provide support for the hypothesis that disrupted redox homeostasis is implicated in HMGB1-associated cellular responses induced by the investigated complexes.

### Modeling the Interactions with TrxR

Given that inhibiting thioredoxin reductase (TrxR) might be responsible (at least partially) for the activity of our compounds, we attempted to gain preliminary insights into their possible interactions with this enzyme by molecular docking. Several of the tested compounds were docked to the redox site of TrxR (PDB accession code: 3EAN^47^) using AutoDock 4.2.^48^ This was meant to model the inhibitor–enzyme complex just before the ligand-exchange reaction taking place. This is why we chose noncovalent docking. Docking results (in terms of the predicted energies) are given in Tables S1–S9. Representative binding poses are given in Figures S1–S16. In most cases, docking yields a few closely lying (in energy) binding poses which can be treated as a population of reasonable solutions. For the best TrxR inhibitor in our set, complex **1f**, the top-scored binding mode (Figure 5A) has the neopentyl-xanthate moiety inserted down into the intersubunit cleft, close to Gly499, Ala26, and Lys29. The gold atom is located at a distance of 3.48 Å from the Se atom of the selenocysteine SEC498. SEC498 is capped from the top by one of the mesitylene aromatic rings, which is predicted to interact also with Ala119, Lys123, and Gly496. The other mesitylene ring is close to the strand of the second subunit, in the proximity of Leu482, Ser483, and Val484.

**Figure 5:**
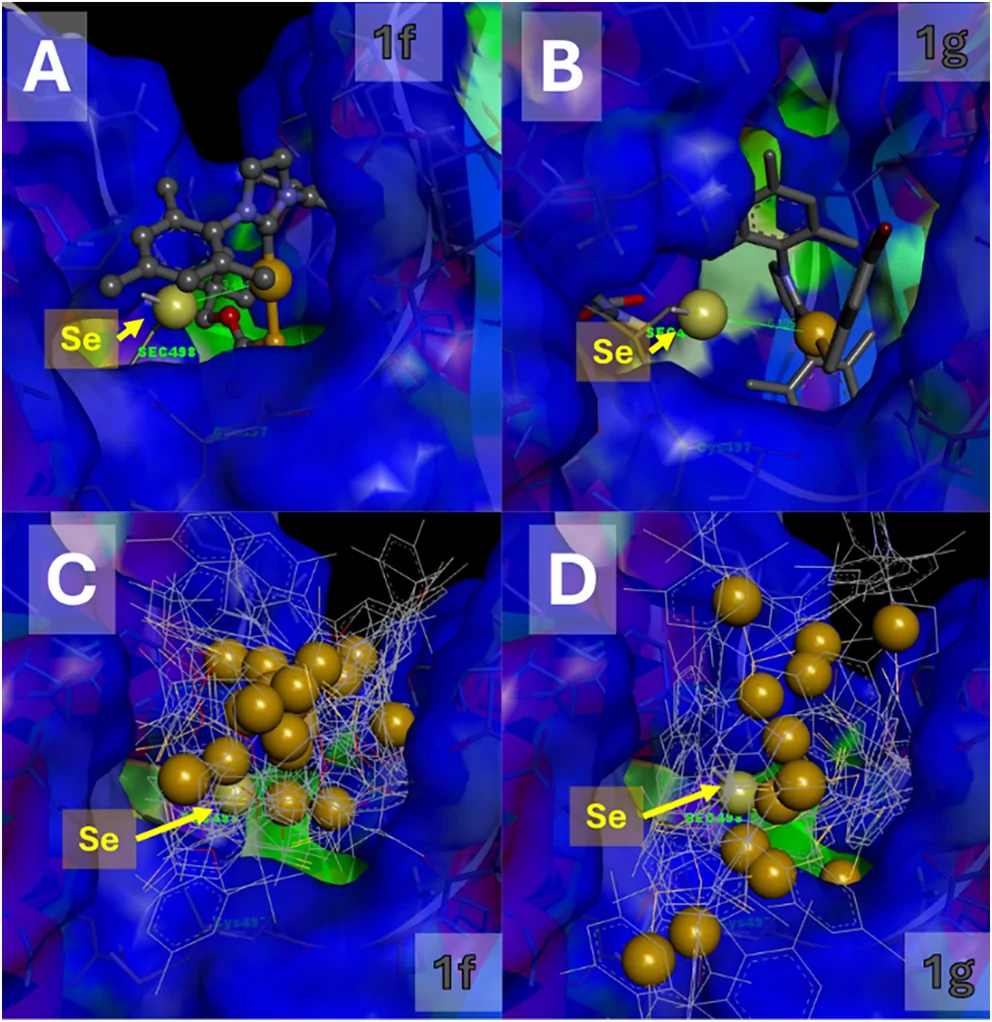
Docking poses for complexes **1f** and **1g**. (A) The top-scored pose for **1f**. (B) The top-scored pose for **1g**. (C) Population of all predicted poses for **1f**. (D) Population of all predicted poses for **1g**. The enzyme is represented as a solvent-accessible surface (blue) with SEC493 and its selenium atom (light yellow ball) represented explicitly. The inhibitors are shown as sticks (A, B) or thin lines (C, D) with the Au atom shown as a golden ball. The C and D panels show that when considering the whole population of generated solutions, the Au atoms of the inhibitors are significantly closer to the Se atom of the enzyme in the case of compound **1f**.

The computed free energies of binding Δ*G* (either the lowest ones or the average of the solutions found) do not correlate significantly with the observed inhibition (Figure 6A). For example, **1g** (almost no experimental inhibition; the best Δ*G* = −5.6 kcal/mol) was predicted to have slightly better Δ*G* values compared to those found for **1f** (the strongest inhibitor; the best Δ*G* = −5.0 kcal/mol).

**Figure 6:**
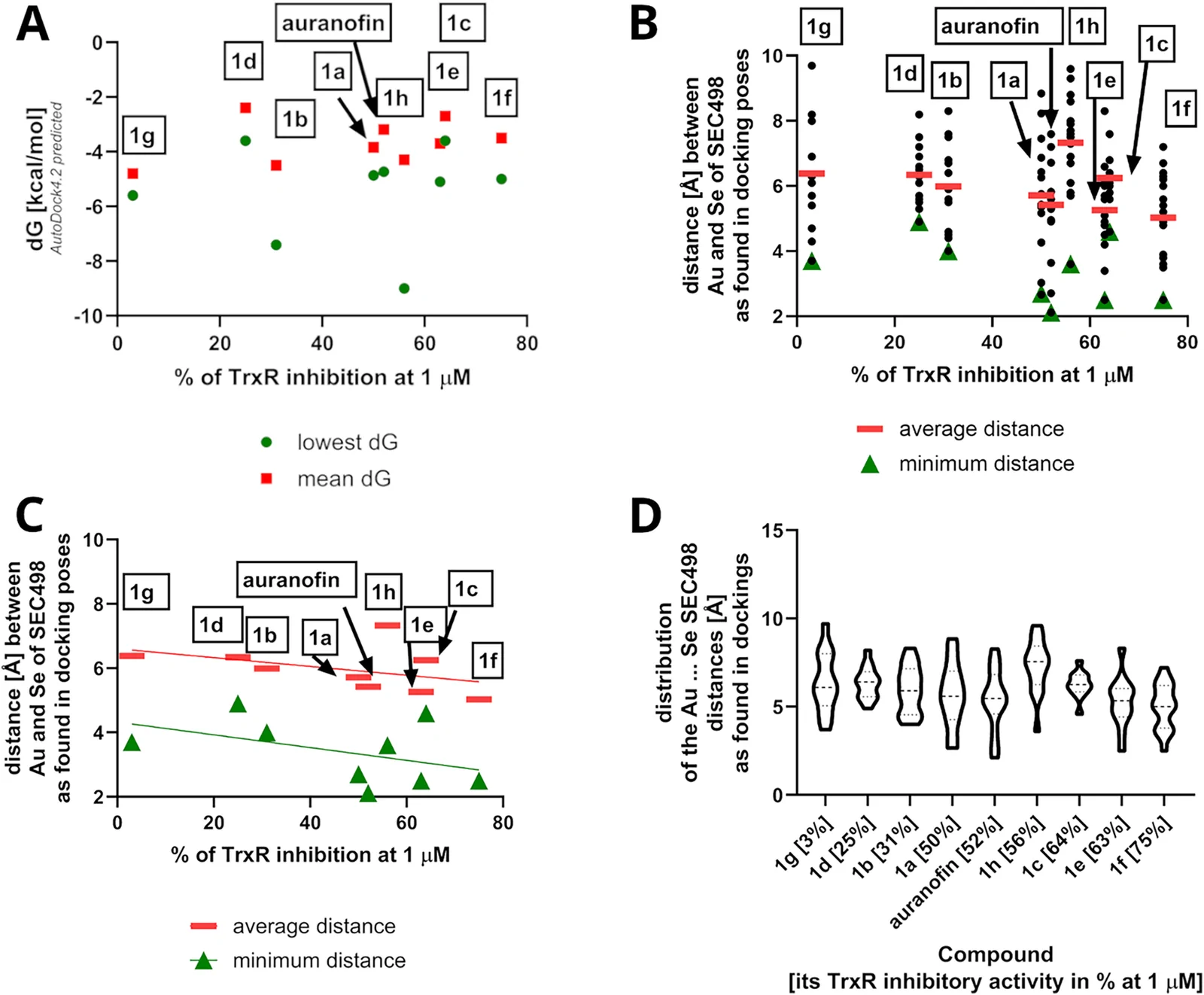
(A) Correlational analysis of the energies found in docking and TrxR inhibition. (B) Correlational analysis of the Au···Se distances found in docking and TrxR inhibition; values from all clusters (black), along with average (red) and minimum (green) distances. (C) Correlational analysis of the Au···Se distances found in docking and TrxR inhibition; only average (red) and minimum (green) distances and regression lines. (D) Correlational analysis of the distributions of the Au···Se distances found in docking and TrxR inhibition.

On the other hand, we spotted an interesting (though weak) correlation linking the experimental inhibition to the distribution of the predicted distances between the inhibitor’s Au atom and the Se atom of the enzyme’s SEC498 (Figure 6B–D). Roughly, the stronger the inhibitor, the shorter the distances between these two key atoms found in the docking poses (all solutions taken into account as a population).

These results could be interpreted as follows. The inhibitory activity in TrxR enzyme is dependent on the reaction, after which the Au atom becomes bound to the Se atom of the selenocysteine SEC498 residue in the enzyme. For this reaction to occur, the inhibitor must be able to place its Au atom close to the Se atom of the enzyme. The docking poses found in the simulations represent probable, relatively stable intermediary configurations on the path of the inhibitors’ approach to this reaction. For weaker inhibitors in our set, docking predicts that many of their possible low-energy complexes with the enzyme would not possess the required close Au···Se positioning (Figure 6B–D, for compound **1g**). In that scenario, even more favorable interaction energies of the intermediate states would not result in (strong) inhibition, since the inhibitor would rarely be close enough for the exchange reaction to happen. In the case of the more active compounds in our set, they are predicted to position their Au atom closer to Se (Figure 6B–D, for compound **1f**). Then, despite less favorable interaction energies of the intermediate states, this should give stronger inhibitory activity. A structural factor that seems to enable this closer Au···Se positioning might be the size/volume of the xanthate and the NHC pieces. Larger compounds, according to the docking, tend to locate slightly further away from the selenocysteine because they are not able to accommodate their bulky pieces in the available space around this key SEC residue. This reasoning yields a working directive for the design of novel compounds according to which sterics at both the NHC and xanthate sites should be tuned to obtain the optimal TrxR inhibition.

It is important to stress the preliminary nature of this docking analysis and its limitations. First of all, we have not performed a validation of the parameters used for modeling Au and Se atoms. Second, the correlations found are weak and are built on just a few examples. Third, we used docking to a rigid enzyme structure, even though flexibility is known to play a role in the TrxR mechanism of action.^49^ Finally, the protocol used was noncovalent docking, thus enabling the modeling of states before the reaction, but it has to be remembered that the inhibition in this case relies on a covalent reaction. All of these limitations will need to be addressed when undertaking a more rigorous approach to modeling the NHC-Au-xanthate complexes with TrxR. Still, a general, qualitative picture of the docked complexes provides a working hypothesis for the design of novel compounds with improved activities.

### Apoptosis and Necrosis Induction

As another step in the mechanistic evaluations, the ability of the tested agents to induce apoptosis and/or necrosis in the treated cells was evaluated to further elucidate the mode of action associated with the observed cytotoxic effects. The results demonstrate that both pathways were engaged (Figure 7), exhibiting significant efficacy. In line with the findings of preceding studies, exposure to metal-based agents, including cisplatin and auranofin, induced apoptosis or necrosis in treated cells, exhibiting a strong correlation with the release of HMGB1.^50,51^ Interestingly, complexes **1a**, **1b**, and **1f**, similar to auranofin, activated both apoptotic and necrotic pathways. In contrast, the remaining investigated compounds predominantly induced apoptosis, indicating that programmed cell death represents the major mechanism underlying their cytotoxic activities.

**Figure 7:**
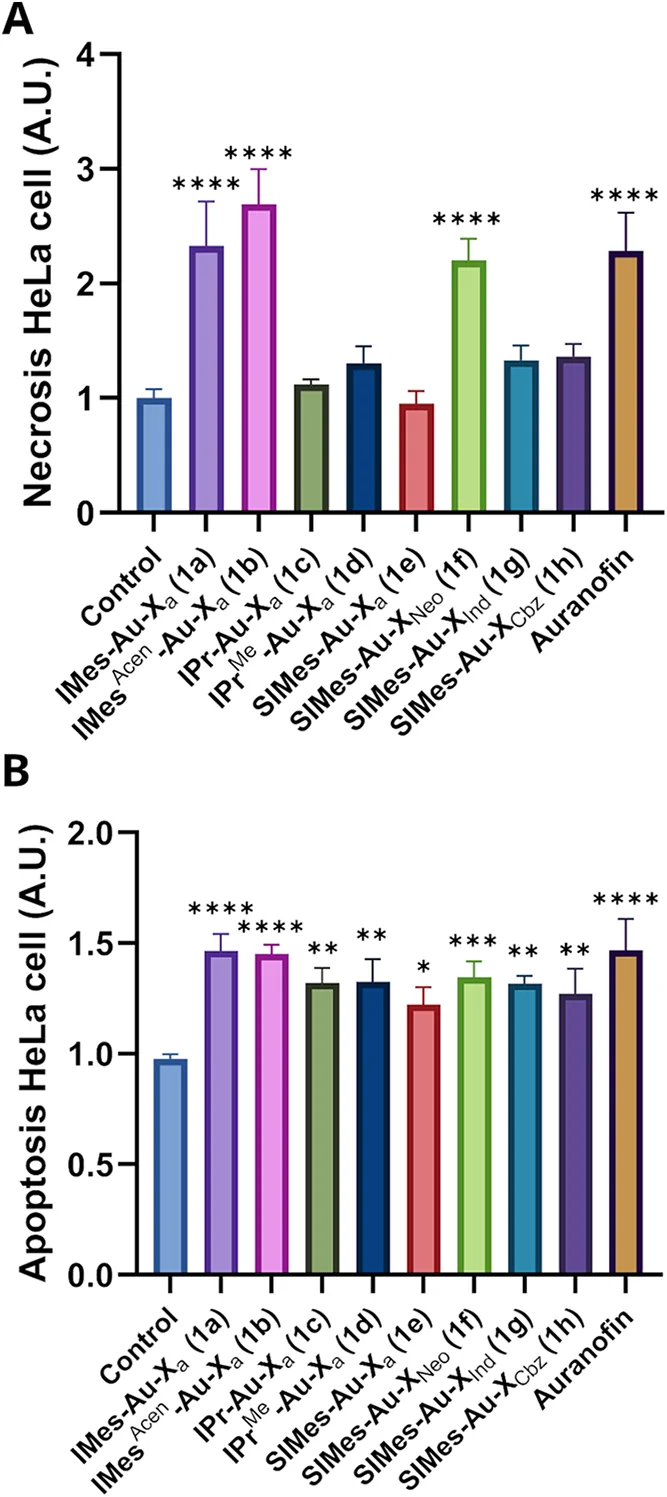
Induction of apoptosis and necrosis in HeLa cells following treatment with NHC-gold-xanthate complexes. Cell death was evaluated using the RealTime-Glo Annexin V Apoptosis and Necrosis Assay (Promega), a 96-well plate-based assay with apoptosis detected by luminescence and necrosis by fluorescence. Panels A and B show fold changes in necrotic (A) and apoptotic (B) cell death, respectively, after 48 h of treatment with NHC-gold-xanthate complexes (1 μM). Statistical significance vs control was marked (*) *p* < 0.05, (**) *p* < 0.01, (***) *p* < 0.001, and (****) *p* < 0,0001. The data presented constitute average results from three measurements ± SD. Error bars represent the standard deviation.

### Intracellular Localization of BODIPY-Labeled Complex

To study the localization and the rate of internalization of our complexes, we synthesized a BODIPY-tagged counterpart **6** of the complex **1e** within three steps (Figure 8H). The synthesis commenced with the alkylation of the NHC precursor **2** with a bromide bearing a BODIPY fluorescent tag, following the protocol developed by Plenio.^52,53^ The respective NHC precursor **4** was subsequently converted into NHC-Au-Cl **5** complexes under the conditions reported by Nolan and Gimeno.^54,55^ Final ion metathesis with xanthate salt (X_a_) in acetone delivered the targeted complexes **6** in high yields (for details, see SI, p. S2).

**Figure 8:**
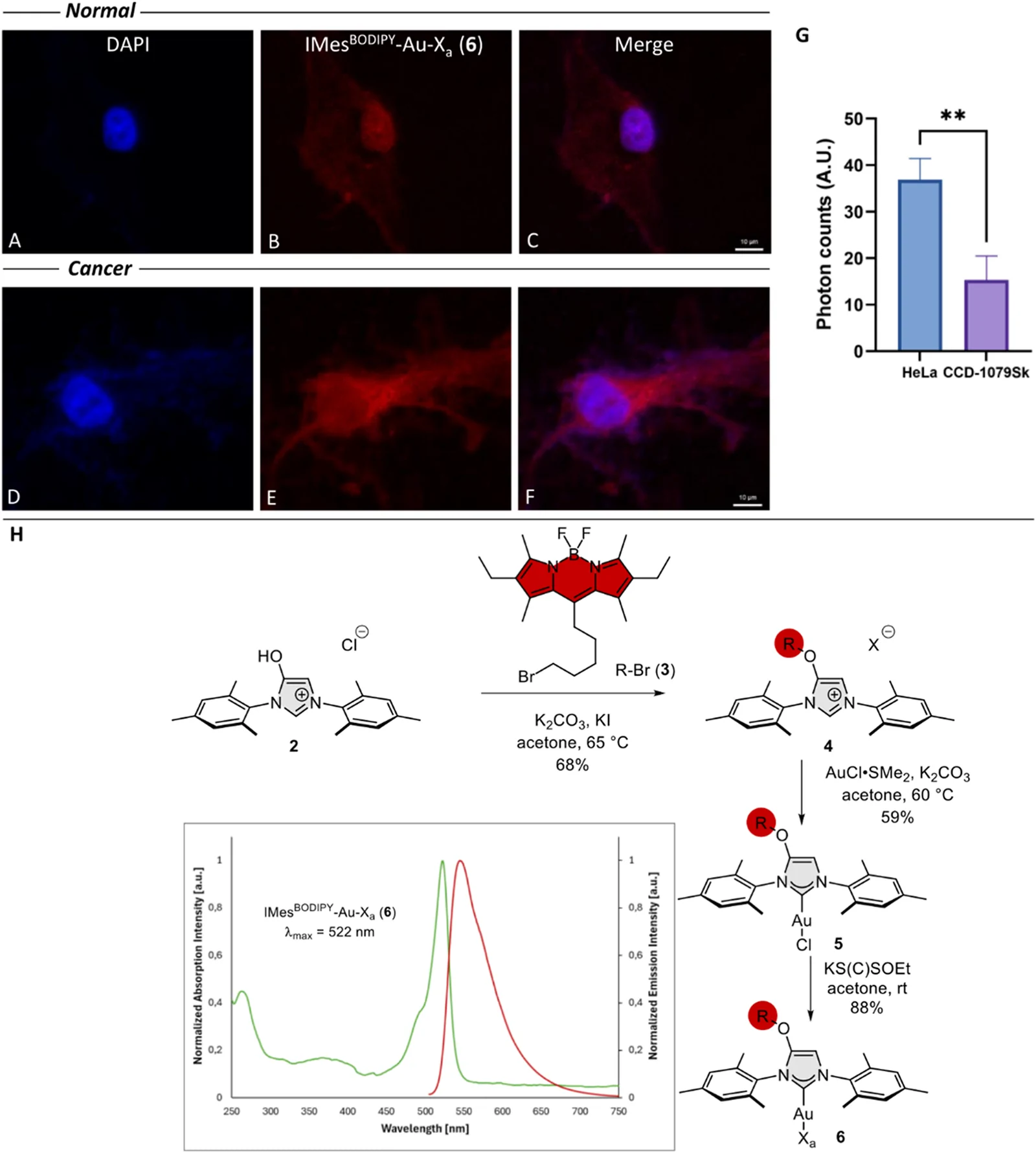
Internalization of BODIPY-labeled NHC-Au-X_a_ complex **6** into human fibroblasts and cervical cancer cells and its synthesis (A–F). Microscopic analysis of BODIPY-labeled NHC-gold-xanthate complex localization into human fibroblasts (A–C) and cervical cancer (D–F) cells. Scale bar 10 μm. (the image has been magnified to a scale of 100×). (G) Difference in photon counts of BODIPY-labeled NHC-Au-X_a_ complex **6** in cervical cancer HeLa and human fibroblast CCD-1079Sk cells. Statistical significance between HeLa cells and human fibroblasts (*) *p* < 0,05. The data presented constitute average results from three measurements ± SD. Error bars represent the standard deviation. (H) Synthetic route to complex **6** and its normalized absorption (green), emission (red); normalized absorbance (blue, λ_max_ = 522 nm ε = 3.47 × 10^4^ M^–1^cm^–1^) and fluorescence (red, excitation at 493 nm, λ_max_ = 544 nm, *c* = 4.2 × 10^–6^ M in DCM) of complex **6**.

To assess the localization and the rate of internalization of our complexes, HeLa cervical cancer cells and CCD-1079Sk normal fibroblast cells were treated for 24 h with BODIPY-tagged complex **6**, which is structurally relevant to complex **1e**. The presence of a fluorescent moiety on the BODIPY-labeled NHC-Au-xanthate complex (excitation at 493 nm, λ_max_ = 544 nm) enabled visualization by confocal microscopy in cells. The localization of the tested complex was studied using confocal/STED microscopy and confirmed by spectrofluorimetric measurement (Figure 8). Results of microscopic analysis illustrated in Figure 8G show a difference in the photon counts of the BODIPY-labeled NHC-Au-X_a_ complex **6** present in cervical cancer (HeLa) and fibroblast cells (CCD-1079Sk). They indicate a higher affinity of the tested complexes for cancer cells, which confirms their selective mode of action. However, because complex **6** contains an additional fluorescent BODIPY moiety, these data should be interpreted as a visualization model for the parent compound rather than direct proof of the identical uptake behavior of complex **1e**.

## Experimental Section

### Cell Culture

The compatibility and anticancer activity of NHC-gold-xanthate complexes were determined against skin fibroblast CCD-1079Sk and cervical cancer cells HeLa obtained from the American Type Culture Collection (Manassas, VA). The cells were cultivated in 96-well plates at a density of 5–7 × 10^3^ cells per well until they reached full confluence in Eagle’s Minimum Essential Medium (EMEM) (ATCC) (for CCD-1079Sk and HeLa), supplemented with 10% fetal bovine serum (FBS) (ATCC), 50 U/mL penicillin, and 50 mg/mL streptomycin (Gibco, Thermo Fisher Scientific, Inc., Waltham, MA) under physiological conditions, at 37 °C with 5% CO_2_.

### Cytotoxicity Studies

#### Neutral Red Assay

In order to assess the cytotoxicity of the tested NHC-gold complexes, a neutral red cytotoxicity assay was used. In brief, after 48 h incubation of cells with the tested agents (0.1–10 μM), a neutral red solution (10 μL/well) was added and incubated for 2 h. Subsequently, the culturing medium was removed, and the cells were fixed for a period of 5 min. In the next step, the fixative solution was discarded, and the acidic solution was added to dissolve the dye. The absorbance was measured at a wavelength of 540 nm using a Varioscan Lux microplate reader (Thermo Fisher Scientific). Values were normalized to the control ± SD.

In order to assess the cytotoxicity of the tested NHC-gold- xanthate complexes, an MTT-based assay has been performed. After 48 h incubation of cancer (HeLa) and normal cells (CCD-1079Sk) with the tested agents at a concentration range of 0, 0.1, 0.5, 1, 5, 10, 50, and 100 μM, the MTT assay protocol was followed as published previously.^56,57^ The absorbance was measured at a wavelength of 570 nm using a Varioscan Lux microplate reader (Thermo Fisher Scientific). Values were described as a percent of control ± SD. The IC_50_ values for treated cells were calculated using nonlinear regression with an [Inhibitor] vs response (three parameters) model in GraphPad Prism 10.1.2 version.

### Hemocompatibility Studies

The experiments were performed according to a previously published protocol.^58^ The collected red blood cells (RBC) obtained from healthy volunteers were suspended in phosphate-buffered saline (PBS) in order to establish a hematocrit of approximately 5%. The tested complexes were used at a concentration of 0.1 – 100 μM and incubated for 1 h at 37 °C. After centrifugation, the relative hemoglobin concentration in the supernatants was spectrophotometrically measured by absorbance (*A*) at a wavelength of 540 nm using a Varioscan Lux microplate reader (Thermo Fisher Scientific). The 0% hemolysis was obtained from samples following the addition of 10 μL of PBS, while the 100% hemolysis was obtained from samples in which 10 μL of 1% Triton X-100 was added to disrupt RBC membranes.

The percentage of hemolysis was calculated according to the following equation:

$$h\mathrm{emolysis}\;\left(\%\right)=\left[\left(A_{‐}\mathrm{sample}-A_{‐}\mathrm{negative}\;\mathrm{control}\right)/\left(A_{‐}\mathrm{positive}\;\mathrm{control}-A_{‐}\mathrm{negative}\;\mathrm{control}\right)\right]\times 100$$

### *K*_i_-67 Assay

The assay was carried out according to the manufacturer’s protocol. In brief, the HeLa cell line (ATCC) was seeded into a 96-well white plate with a transparent bottom (80 μL/well). After 24 h, cells were exposed to the tested complexes at concentrations in the IC_50_ range: 0.5–10 μM and incubated for 48 h at 37 °C and 5% CO_2_. After that, 5X Lumit Lysis Buffer II was added (20 μL/well) to each well, and then the plate was incubated with shaking for 40 min at 800 rpm. Following incubation, 2X Anti-hK_i_-67 mAb antibody mixture (100 μL) was added and incubated for 90 min. Finally, Lumit Detection Substrate C (50 μL/well) was added and incubated for 5 min at room temperature, and then luminescence was measured using a Varioscan Lux plate reader (Thermo Fisher Scientific).

### Mode of Action

#### Apoptosis/Necrosis Detection

The engagement of apoptotic and necrotic pathways in NHC-Au-X complex-induced cell death was evaluated using the RealTime-Glo Annexin V Apoptosis and Necrosis Assay (Promega), a nonflow cytometric, 96-well plate-based multiplex assay that enables simultaneous luminescent detection of apoptosis and fluorescent detection of necrosis. The HeLa cells were exposed to the tested complexes at a concentration of 1 μM for 48 h at 37 °C and 5% CO_2_. In addition, cells were treated with auranofin at concentrations of 1 and 5 μM, used as a positive control. Finally, an equal volume of detection reagent (100 μL) was added to each well, and apoptosis-related luminescence signals (Annexin V detection) and necrosis-related fluorescence signals (λex = 485 nm, λem = 530 nm) were recorded using a Varioscan Lux plate reader (Thermo Fisher Scientific).

#### HMGB1 Assay

The Lumit HMGB1 Immunoassay (Promega) was used for detecting High Mobility Group Box 1 (HMGB1) protein released from HeLa cells after treatment with the tested complexes or auranofin (1 μM). After 24 h, 5× Anti-hHMGB1 antibody mixture (20 μL) was added to wells containing cultured cells or HMGB1 (Human) Positive Control dilutions and incubated for 60 min. Then, 5× Lumit Detection Reagent B was added to each assay well of the plate, shaken, and incubated for 5 min at room temperature. Finally, luminescence was measured using a Varioscan Lux plate reader (Thermo Fisher Scientific).

#### Measurement of ROS Formation

The ROS-Glo H_2_O_2_ Assay (Promega) was used to assess the level of reactive oxygen species (ROS) in cervical cancer HeLa cells exposed to NHC-gold-xanthate complexes or auranofin (0.5 μM). The assay was performed in a 96-well plate format. ROS-Glo H_2_O_2_ substrate was added to cell cultures at the end of the treatment and reacted with H_2_O_2_ present in our samples. The reaction product, a luciferin precursor, was converted to luciferin by adding ROS-Glo Detection Solution. The luminescence was measured with the use of a microplate reader (Varioscan Lux, Thermo Fisher Scientific). The data were normalized to the bioluminescent signal intensity of untreated cells and normalized to the control.

#### TrxR Inhibition Assay

The thioredoxin reductase (TrxR) inhibition assay was performed using the Thioredoxin Reductase Colorimetric Assay Kit (Cat. No. 10007892, Cayman Chemical) with minor modifications. The assay was performed using recombinant human thioredoxin reductase 1 (TrxR1; Cat. No. 39624, Cayman Chemical). The tested compounds were incubated with recombinant TrxR1 enzyme for 30 min at room temperature before initiation of the enzymatic reaction. The assay mixture was prepared in a 96-well plate in duplicate according to the manufacturer’s protocol. Briefly, test wells contained TrxR assay buffer, recombinant TrxR1 enzyme (20 μL), the tested compounds (1 μM), DTNB, and NADPH in a final volume of 200 μL. The enzymatic reaction was initiated by adding 20 μL of NADPH and 20 μL of DTNB. The plate was shaken for 30 s at 400 rpm, and absorbance was measured at 405 nm using a Varioskan Lux plate reader (Thermo Fisher Scientific). The TrxR inhibitory effect was expressed as a decrease in the absorbance change rate at 405 nm and normalized to the untreated enzyme control.

### Intracellular Localization of BODIPY-Labeled Complexes

Cancerous (HeLa) and normal cells (CCD-1079Sk) were seeded into six-well plates on which coverslips had been placed previously. After a 24-h incubation period at 37 °C and 5% CO_2_, the NHC-Au-X_a_ complexes bearing the BODIPY fluorescent tag were added and incubated for an additional 24 h under the same conditions at a concentration of 1 μM.

#### DAPI Staining

After removing the medium, the wells were rinsed with PBS (Corning) and fixed with 4% PFA (Sigma-Aldrich) for 10 min. The cells were rinsed three times with PBS, and then 0.1% Triton X-100 (Sigma-Aldrich) was added for 5 min to permeabilize the membrane. The wells were then rinsed three times with PBS, after which a solution of DAPI (3 μg/mL) (Sigma-Aldrich) was added. The slides were incubated for five to 10 min at room temperature, protected from light. After staining, the slides were rinsed three times with PBS to remove excess dye. Finally, the prepared coverslips were placed on the basal slides with a drop of mounting medium (Abberior MOUNT). The edges of the slides were sealed with clear varnish.

#### Microscopic Evaluation

Fluorescent images were acquired using an Abberior STEDYCON super-resolution microscope equipped with a 100x oil immersion objective (NA 1.4). The nucleus was stained with DAPI ex 405 nm, em 415–480 nm and localization of the BODIPY-labeled NHC-Au-X_a_ complexes **6** was detected using excitation at 490 nm and emission at 560 nm.

#### Molecular Modeling

The enzyme structure for docking (PDB identifier: 3EAN^47^) was downloaded from the PDB database.^59^ Water molecules were removed, and hydrogen atoms were added. The structure was preprocessed in AutoDock Tools.^48^

The grids were calculated with AutoGrid 4.^48^ The following line was added to the default AutoDock 4 parameter file for the sake of modeling the Au atom: “atom_par Au 3.29 0.039 12.000 -0.00110 0.0 0.0 0 -1 -1 1”. The docking box was centered on SEC498 and its size was 60 × 60 × 60 points (with one point per 0.375 Å spacing). All receptor residues were rigid.

The inhibitors’ structures for docking were taken from their single-crystal structures from our previous work,^30^ deposited in the Cambridge Structural Database.^60^ The entries used were **1e**-YULDUI, **1f**-YULFAQ, **1g**-YULGAR, **1h**-YULGUL, and **1b**-YULHEW. Structures for 1c and 1d were prepared manually based on the YULFIY entry. Similarly, the structure for **1a** was prepared manually based on the YULHEW structure. For modeling auranofin, the AGLPAU structure was used. The ligands’ torsional freedom was allowed, except for the −S–C­(=S)–, −C­(=S)–O– torsions that were constrained to the flat geometry.

The docking procedure (noncovalent docking) used the genetic algorithm with the parameters as follows: external grid energy (extnrg) of 1000.0 kcal/mol, 150 individuals in population (ga_pop_size), 2500000 maximum energy evaluations (ga_num_evals), 27000 maximum number of generations (ga_num_generations), 1 top individual surviving to the next generation (ga_elitism), 0.02 gene rate mutation (ga_mutation_rate), 0.8 crossover rate (ga_crossover_rate), window size of 10 generations (ga_window_size), and 0.0 and 1.0 for α and β parameters of Cauchy distributions. Twenty global searches (do_global_only) were done for each ligand. The structures resulting from the search were then clustered. For qualitative assessment of the binding energy, both the lowest and mean energies of the clusters were collected and considered. Molecular graphics were prepared in Biovia Discovery Studio Visualizer.^61^

### Statistics

Data represent duplicates or triplicates and are expressed as the mean ± SEM. Differences were considered significant if *p* < 0.05. GraphPad Prism 10.1.2 (GraphPad Software, La Jolla, CA) allowed for statistical analysis of data using Student’s *t* test (for independent samples) to compare two groups, or one-way ANOVA followed by Tukey’s multiple comparison test to compare more than two groups within an experiment.

## Conclusions

In this study, we demonstrated that exposure of cells to NHC-Au-xanthate complexes leads to a significant increase in ROS and DAMPs, including the HMGB1 protein levels, as well as the induction of both apoptosis and necrosis (Figure 9). These results suggest that the cytotoxic response of cells to the examined agents is primarily mediated by oxidative stress, as an effect of TrxR inhibition, which results in an intracellular redox imbalance and the activation of distinct cell death pathways. These mechanisms are consistent with the previously reported effects of other metal-based agents, such as auranofin and cisplatin. Docking studies provided preliminary support for TrxR as a plausible target of the investigated NHC-Au-xanthate complexes, suggesting that inhibitory activity depends predominantly on the ability of the compound to position the Au atom close to the selenocysteine residue of TrxR. The most active compounds were predicted to form poses with shorter Au···Se distances, whereas weaker inhibitors more often adopted low-energy poses that were geometrically less favorable for ligand exchange. Although these correlations are preliminary and require validation with more rigorous modeling, they provide a useful working hypothesis that steric tuning of both the NHC and xanthate ligands may improve TrxR inhibition. Higher accumulation of the BODIPY-labeled NHC-Au-xanthate complex in cervical cancer cells than in normal fibroblasts supports preferential uptake or retention for this group of compounds. Among the investigated compounds, complex SIMes-Au-X_Neo_ (**1f**) emerged as the most attractive lead candidate. Its potent antiproliferative activity was accompanied by pronounced selectivity toward cancer cells, negligible hemolytic activity, efficient TrxR inhibition, enhanced ROS generation, and increased HMGB1 release. Furthermore, similarly to auranofin, complex **1f** induced both apoptosis and necrosis, suggesting activation of multiple cell death pathways. Collectively, these features point to a mechanism involving disruption of redox homeostasis, TrxR inhibition, ROS accumulation, and DAMP-associated signaling and highlight the potential of complex **1f** for further development as a gold-based anticancer agent with possible chemoimmunotherapeutic relevance. Overall, the findings reported herein provide valuable insights into the molecular basis of Au-based agents, especially NHC complexes, and support their potential as therapeutic agents targeting neoplastic cells through redox-dependent and immunogenic stress responses.

**Figure 9:**
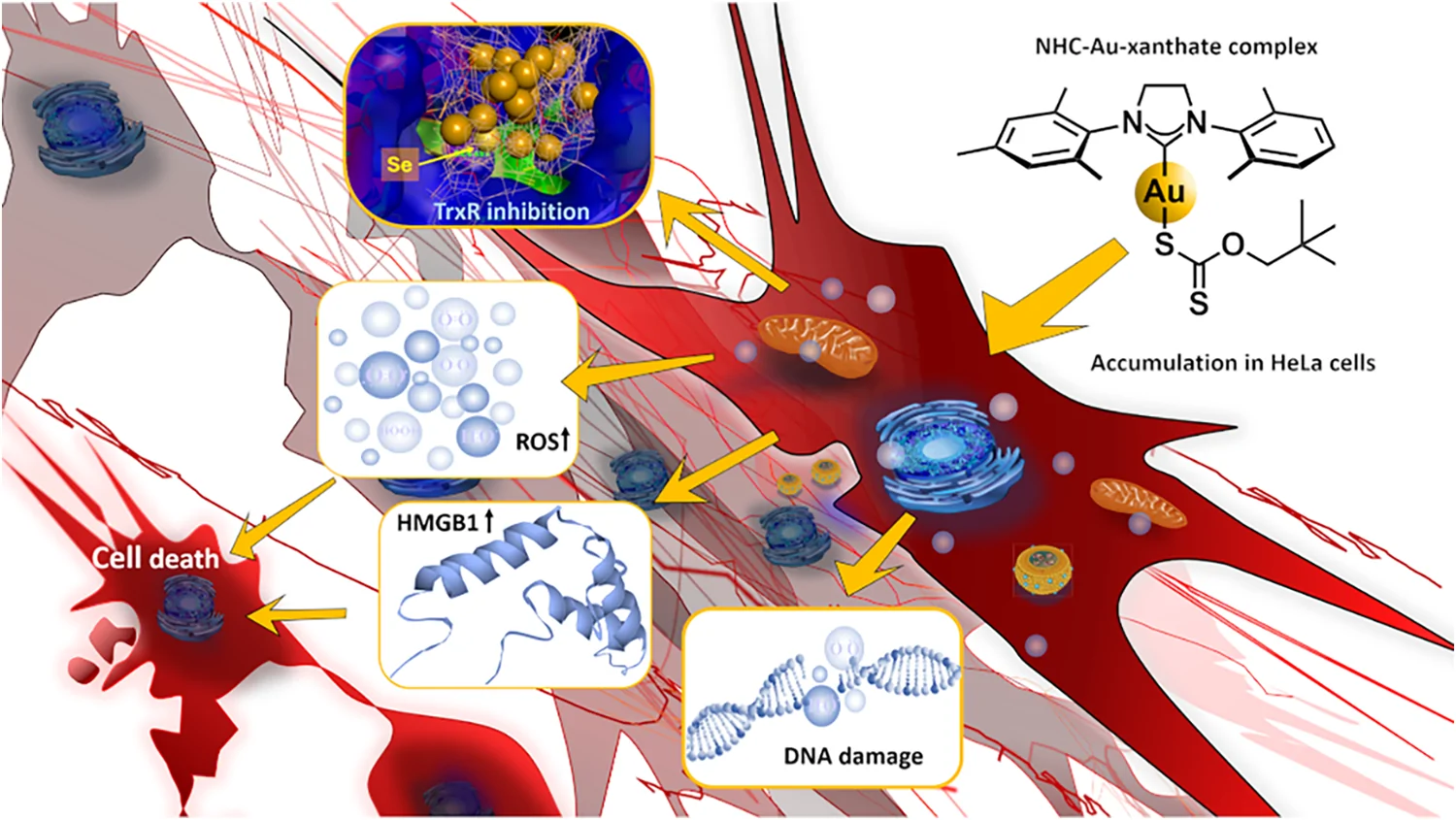
Proposed mechanism involves selective intracellular accumulation of complex 1**f**, followed by DNA damage and TrxR inhibition, resulting in the disruption of redox homeostasis and increased ROS generation. These events promote apoptotic/necrotic cell death, accompanied by HMGB1 release and DAMP-associated signaling, potentially contributing to immunogenic cellular responses. Illustration by Agnieszka Z. Wilczewska, used with permission.

## Supplementary Material



## Notes

Experimental procedures, NMR spectra, and molecular docking details. (PDF)
