# Supplementary material for: NHC-Au-Xanthate Complexes: Selective Cytotoxic Activity and Mechanistic Insights
Source: ACS Omega. 2026 Oct 12;11(42):65351–63. doi: 10.1021/acsomega.6c07778 (PMC13718240; doi:10.1021/acsomega.6c07778)
Supplement: Supplementary file 1 [file ao6c07778_si_001.pdf]

NHC-Au-Xanthate complexes: selective cytotoxic activity and mechanistic insights

Katarzyna Niemirowicz-Laskowska,*[a] Marta Pietruszyńska,[b] Supratim Chakraborty,[c] Piotr F. J. Lipiński,[d] Wioletta Kośnik,[e] Beata Skonieczna,[a] Iwona Misztalewska-Turkowicz,[f] Halina Car,[a,b] and Michał Michalak,*[c]

[a] Department of Experimental Pharmacology Medical University of Białystok, Szpitalna 37, 15-295 Bialystok

[b] Department of Clinical Pharmacology, Medical University of Bialystok, Waszyngtona 15A, 15-274 Bialystok, Poland

[c] Institute of Organic Chemistry, Polish Academy of Sciences, Kasprzaka 44/52, 01-224 Warsaw

[d] Department of Neuropeptides, Mossakowski Medical Research Institute, Polish Academy of Sciences, Pawinskiego 5, 02-106 Warsaw

[e] NanoVelos S.A., Rakowiecka 36, 02-532 Warsaw, Poland

[f] Faculty of Chemistry, University of Bialystok, Ciolkowskiego 1K, Bialystok, Poland

* corresponding authors: katarzyna.niemirowicz-laskowska@umb.edu.pl

michal.michalak@icho.edu.pl

Table of contents

1. Experimental section 2
2. Synthesis of NHC-Au-X complexes 2
3. Molecular docking results 5
4. Copies of spectra 23
5. References 30

1. Experimental section

General Remarks

NMR spectra were recorded in $CDCl_3$ or CD_2Cl_2 solutions; chemical shifts are quoted on the δ scale, ppm, with the solvent signal as the internal standard ($CHCl_3$, ^{1}H NMR 7.26 ppm; $CDCl_3$, ^{13}C{1H} NMR 77.0 ppm, CD_2Cl_2 ^{1}H NMR 5.32 ppm; ^{13}C{1H} NMR 54.0 ppm. High resolution mass spectra (HR MS) were taken using EI technique or electrospray ionization (ESI). Column chromatography was performed on Merck silica gel 60, 230-400 mesh. TLC was performed on aluminum sheets, Merck 60F 254. Anhydrous solvents were obtained by distillation over $CaCl_2$ (DCM) or $CaSO_4$ (acetone). Organic extracts were dried over Na_2SO_4 and solvents were evaporated on a rotary evaporator. Reagents were used as they were purchased unless otherwise indicated. AuCl•SMe_2 was prepared according to the literature procedure.[1] Xanthate salt KS(C)SOEt was received from Sigma Aldrich, and precipitated from a mixture acetone/petroleum ether before use. Complexes **1a-1h** were synthesized following literature procedure and the purity of the investigated complexes were confirmed by elemental analysis.[2]

2. Synthesis of NHC-Au-X complexes

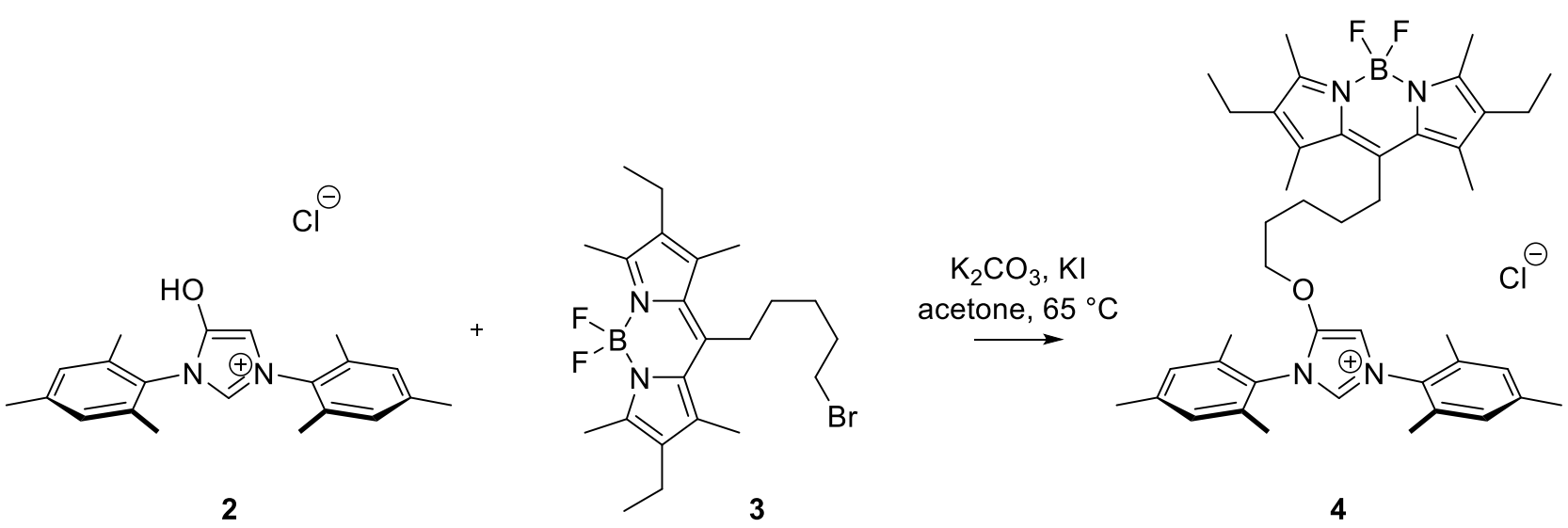

NHC precursor 4 was synthesized according to modified lit. procedure.[3] 1,3-bis(2,4,6-Trimethylphenyl)-5-hydroxy-1H-imidazol-3-ium chloride[4] **2** (200.2 mg, 0.56 mmol), bromide[5] **3** (327.3 mg, 0.57 mmol, 1.5 equiv.), K_2CO_3 (154.9 mg, 1.12 mmol, 2.0 equiv.), and KI (279.1 mg, 1.68 mmol, 3.0 equiv.) were placed in 7 mL vial and dry acetone (4.0 mL) was added. The resulting suspension was heated at 65 °C (temp. of oil bath), cooled to rt and solvent was evaporated. The residue was redissolved in DCM, and filtered through pad of Celite. Then solvent was evaporated, and residue was chromatographed on silica to give (10-20%

acetone/DCM) to give salt **3** as a dark-red solid (280.4 mg, 68%). ^{1}H NMR (400 MHz, $CDCl_3$) δ 9.00 (d, *J* = 1.9 Hz, 1H), 7.43 (d, *J* = 1.9 Hz, 1H), 7.03 – 6.99 (m, 4H), 4.42 (t, *J* = 6.2 Hz, 2H), 2.95 – 2.86 (m, 2H), 2.46 (s, 6H), 2.31 - 2.34 (m, 2H) overlapping 2.38 (q, *J* = 6.7 Hz, 4H), 2.33 (s, 3H), 2.32 (s, 3H), 2.24 (s, 6H), 2.21 (s, 7H), 2.10 (s, 5H), 1.80 (t, *J* = 6.9 Hz, 2H), 1.57 – 1.50 (m, 2H), 1.02 (t, *J* = 7.5 Hz, 6H). Spectral data are in agreement with those reported.[3]

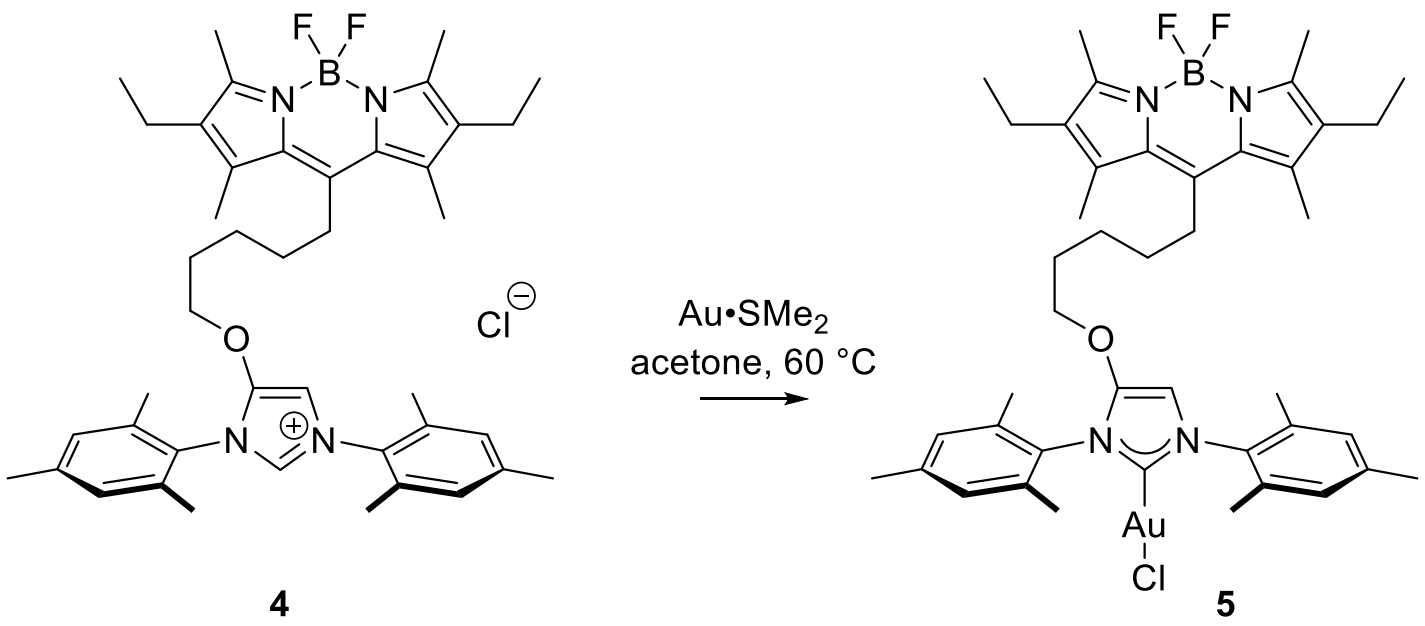

Complex 5 was obtained according to modified lit. procedure.[3] A 20 mL vial was charged with NHC precursor **4** (265.2 mg, 365 μmol), dried K_2CO_3 (50.2 mg, 363 μmol) and AuCl•SMe_2 (107.1 mg, 363 μmol), and dry acetone (16 mL) was added. The resulting suspension was heated at 60 °C (temp. of oil bath) for 16 h, cooled to rt, filtered through syringe filter, and evaporated. The residue was chromatographed on silica (2% acetone/hexanes) to give complex **5** as deep-red oil which was dissolved in minimal volume of DCM and crashed with EtOAc to give deep-red solid (198.3 mg, 59%). ^{1}H NMR (400 MHz, CD_2Cl_2) δ 7.05 (d, *J* = 3.8 Hz, 4H), 6.40 (s, 1H), 4.01 (t, *J* = 6.2 Hz, 2H), 2.98 – 2.89 (m, 2H), 2.45 (s, 6H), 2.44 – 2.48 (m, 2H) overlapping 2.42 (q, *J* = 7.6 Hz, 2H), 2.37 (s, 3H), 2.36 (s, 3H), 2.27 (s, 6H), 2.18 (s, 6H), 2.12 (s, 6H), 1.80 – 1.68 (m, 2H), 1.64 – 1.44 (m, 2H), 1.05 (t, *J* = 7.6 Hz, 6H). The spectroscopic data are in agreement with those reported.[3]

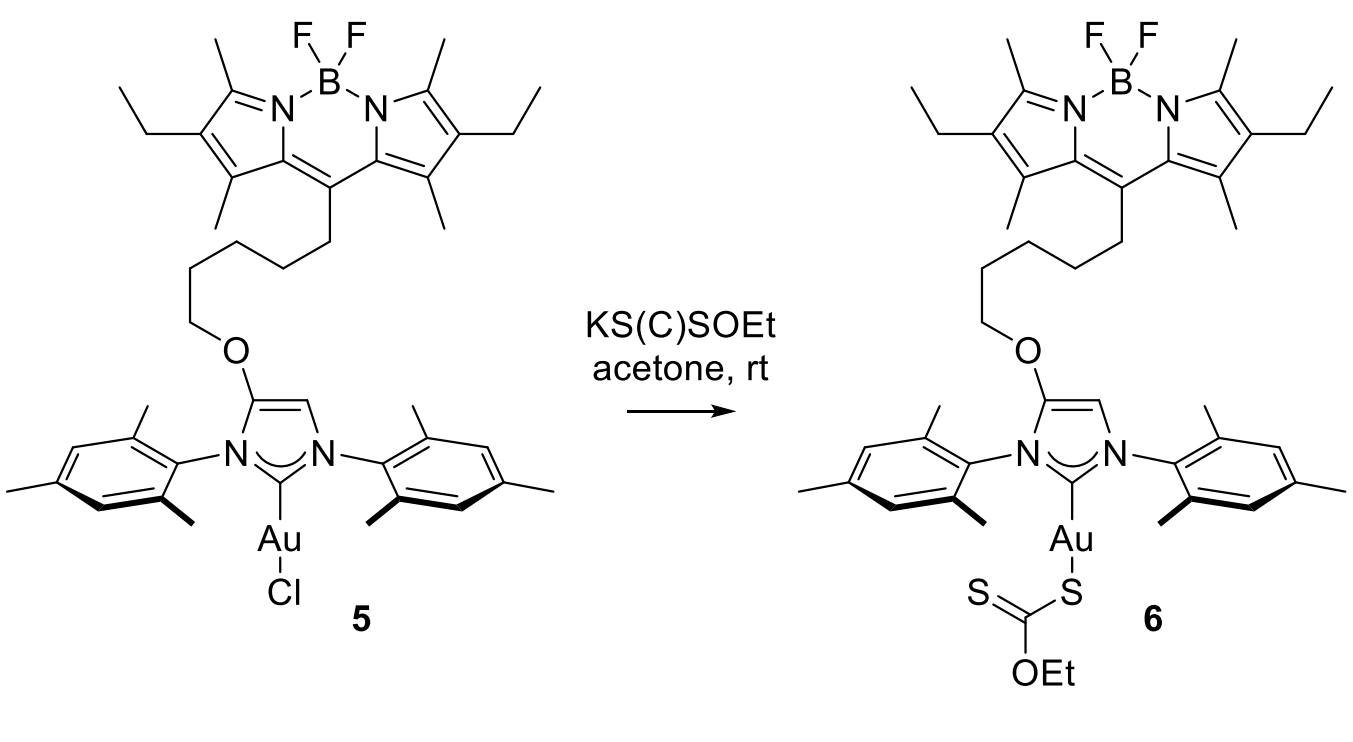

Complex 6 To a solution of NHC-Au-Cl **5** (186.3 mg, 201 μmol) in anhydrous acetone (16 mL), KSC(S)OEt (32.3 mg, 201 μmol, 1.0 equiv.) was added and stirred for 16 h at rt. Then reaction mixture was passed through syringe filter, evaporated, and the oily residue was treated with *n*-pentane (6 mL), and stirred for additional 15 min. Thus solid formed was filtered, washed with *n*-pentane (2 × 10 mL), and dried in vacuo to give complex **6** as a deep-red solid (179.7 mg, 88%). mp >175 °C (DCM/*n*-pentane); ^{1}H NMR (400 MHz, CD_2Cl_2) δ 7.05 (d, *J* = 3.8 Hz, 4H), 6.44 (s, 1H), 4.28 (q, *J* = 7.1 Hz, 3H), 4.03 (t, *J* = 6.1 Hz, 2H), 2.99 – 2.89 (m, 2H), 2.46 (s, 7H), 2.45 – 2.49 (m, 2H) overlapping 2.42 (q, *J* = 7.6 Hz, 2H), 2.38 (s, 3H), 2.36 (s, 3H), 2.29 (s, 6H), 2.20 (s, 6H), 2.15 (s, 6H), 1.81 – 1.73 (m, 2H), 1.64 – 1.44 (m, 2H), 1.17 (t, *J* = 7.1 Hz, 3H), 1.07 (t, *J* = 7.5 Hz, 6H); ^{13}C NMR (100 MHz, CD_2Cl_2) δ 225.1, 177.1, 152.5, 148.3, 144.8, 140.2, 140.1, 136.2, 135.9, 135.9, 135.4, 133.2, 131.6, 131.2, 129.6, 129.6, 99.2, 73.0, 69.4, 31.6, 28.8, 28.7, 26.7, 21.3, 18.1, 17.9, 17.5, 15.0, 14.2, 13.6, 12.6; HR MS (TOF AP) calcd for $C_{46}H_{60}AuBF_2N_4O_2S_2$ $[M+H]^+$: 1011.3963; found: 1011.3959. Normalized absorbance (blue, λ_{max} = 522 nm ε = 3,47 ×10^4 $M^{-1}cm^{-1}$) and fluorescence (red, excitation at 493 nm, λ_{max} = 544 nm, c = 4.2 × 10^{-6} M in DCM) of complex **6**.

3. Molecular docking results

Table S1. Docking results for compound **1b.**

Pose cluster #	Estimated Free Energy of Binding [kcal/mol]	Final Intermolecular Energy [kcal/mol]	Distance between Au and Se of SEC498 [Å]
1	-7.4	-8.9	6.4
2	-5.2	-6.7	5.9
3	-4.6	-6.1	4.0
4	-4.6	-6.1	4.4
5	-4.6	-6.1	4.6
6	-4.5	-6.0	6.7
7	-4.5	-6.0	5.5
8	-4.4	-5.9	5.6
9	-4.2	-5.7	4.5
10	-4.1	-5.6	8.3
11	-4.0	-5.5	6.8
12	-3.7	-5.2	7.6
13	-3.2	-4.7	7.5

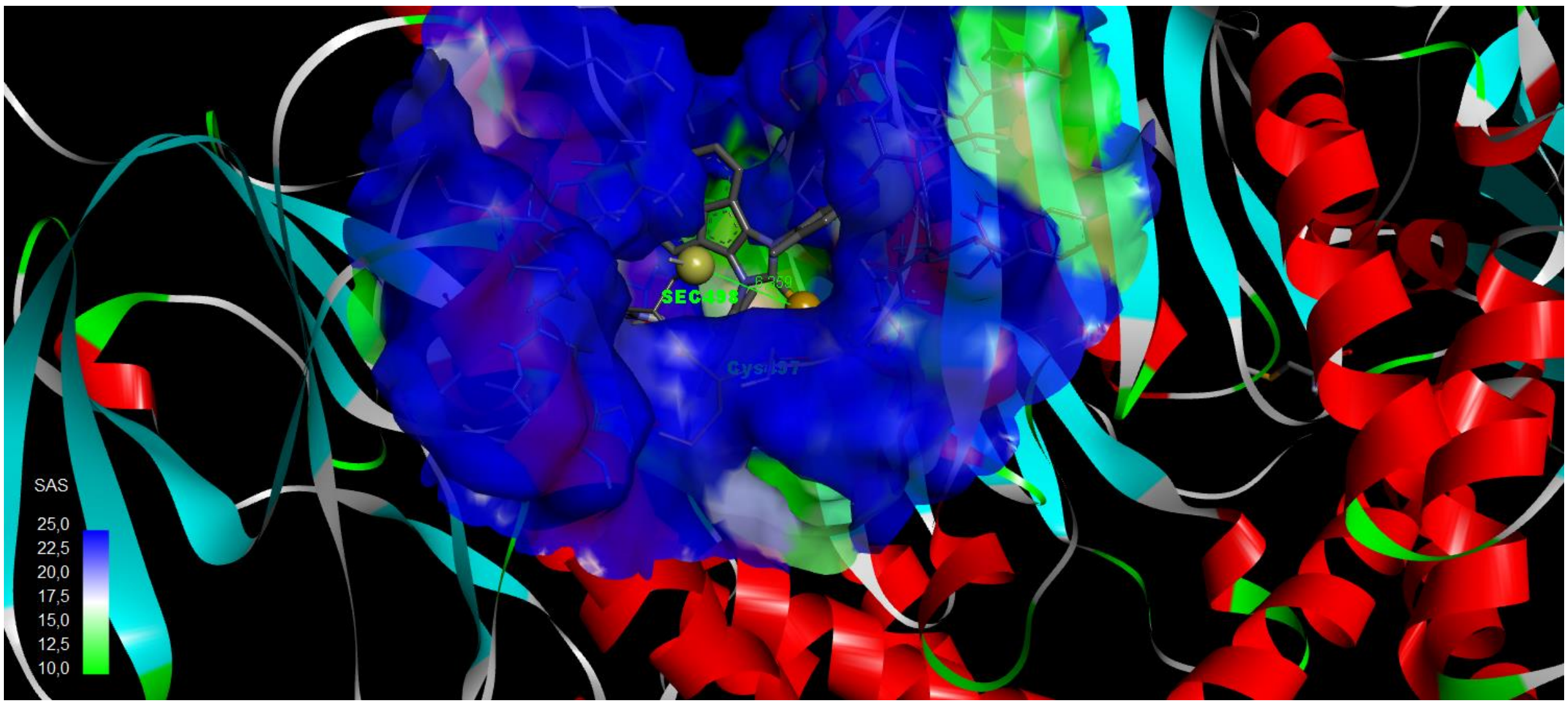

Figure S1. Overview of the top-scored binding pose for compound **1b.**

The enzyme is represented as a solvent-accessible surface around the active site (blue), ribbons depicting secondary structures, with SEC493 and its selenium atom (light yellow ball) represented explicitly. The inhibitor is shown as sticks with the Au atom depicted as a golden ball.

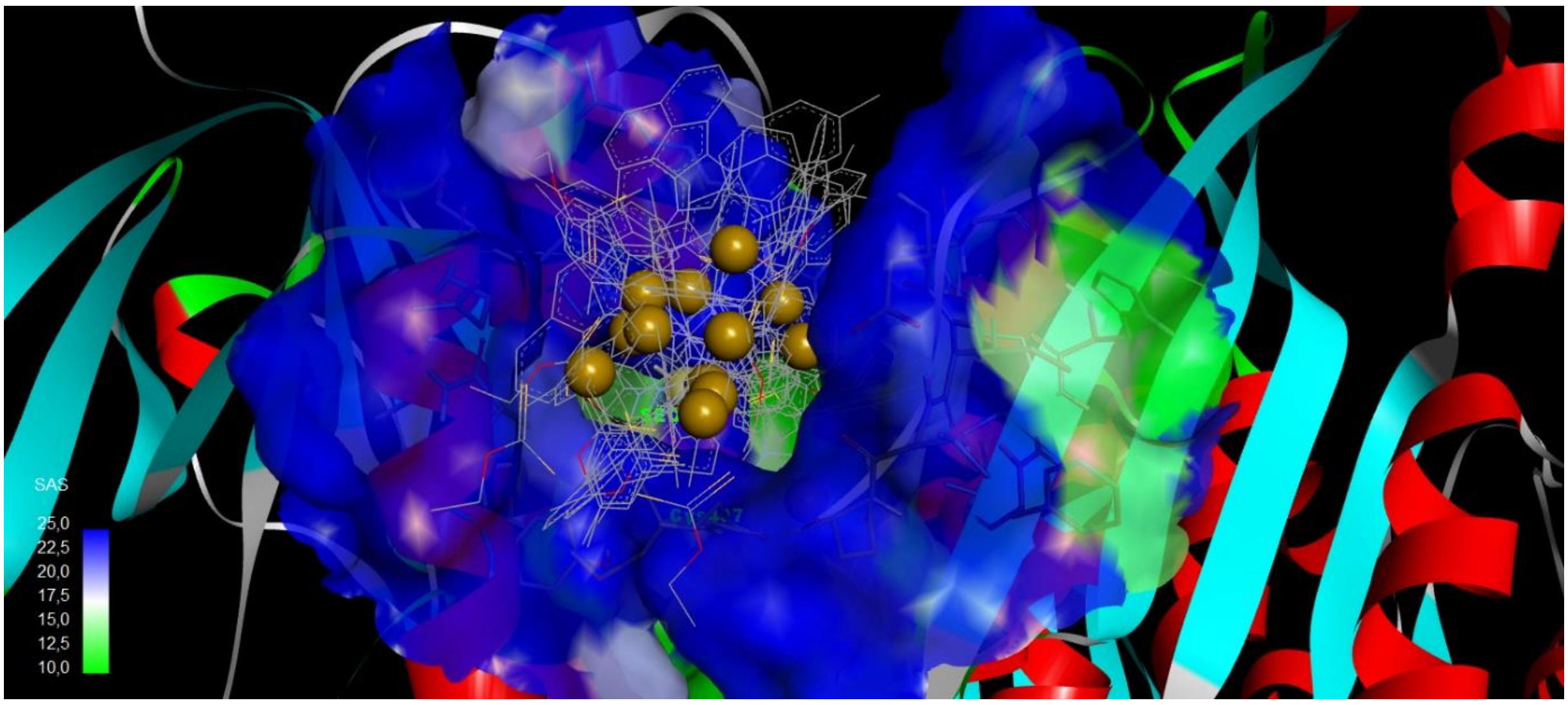

Figure S2. Population of all solutions found for **1b.**

The enzyme is represented as a solvent-accessible surface around the active site (blue), ribbons depicting secondary structures, with SEC493 and its selenium atom (light yellow ball) represented explicitly. The inhibitor is shown as thin lines with the Au atom depicted as a golden ball.

Table S2. Docking results for compound **1f.**

**Pose cluster #**	**Estimated Free Energy of Binding [kcal/mol]**	**Final Intermolecular Energy [kcal/mol]**	**Distance between Au and Se of SEC498 [Å]**
1	-5.0	-6.8	3.5
2	-4.8	-6.6	3.5
3	-4.8	-6.6	2.5
4	-3.9	-5.7	3.8
5	-3.9	-5.7	3.6
6	-3.7	-5.5	3.9
7	-3.7	-5.5	7.2
8	-3.6	-5.4	4.9
9	-3.5	-5.3	6.0
10	-3.3	-5.0	4.8
11	-3.2	-5.0	5.0
12	-3.2	-5.0	5.6
13	-3.1	-4.9	5.4
14	-3.1	-4.9	6.2
15	-3.1	-4.9	6.3
16	-3.1	-4.9	5.0
17	-2.9	-4.7	5.2
18	-2.6	-4.4	6.4
19	-2.2	-4.0	6.7

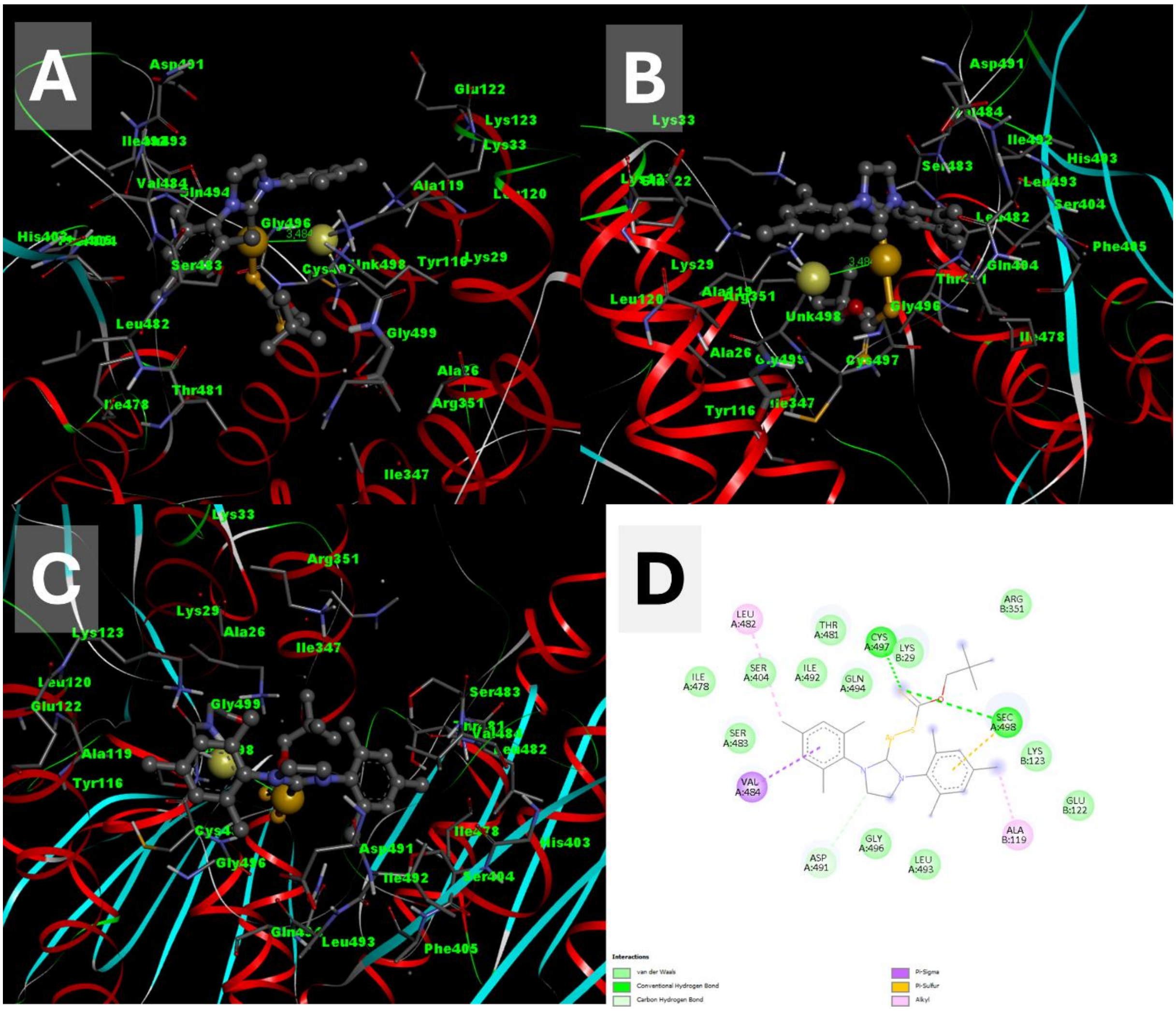

Figure S3. Top-scored binding pose for compound **1f.**

A, B, C. Different views of the binding mode. **D.** Schematic representation of the interactions.

The enzyme is represented as ribbons depicting secondary structure. The residues closer to the inhibitor are depicted explicitly as thin sticks. The selenium atom of SEC498 is shown as a light yellow ball). The inhibitor is shown as balls and sticks with the Au atom shown as a thick, golden ball. Several side chains of the enzyme are labelled with green text.

Table S3. Docking results for compound **1c.**

**Pose cluster #**	**Estimated Free Energy of Binding [kcal/mol]**	**Final Intermolecular Energy [kcal/mol]**	**Distance between Au and Se of SEC498 [Å]**
1	-3.6	-6.3	6.8
2	-3.5	-6.2	6.8
3	-3.3	-6.0	6.1
4	-3.0	-5.7	6.2
5	-2.9	-5.6	6.8
6	-2.8	-5.5	5.6
7	-2.7	-5.4	7.6
8	-2.5	-5.2	6.4
9	-2.2	-4.9	4.6
10	-2.1	-4.8	6.0
11	-2.0	-4.7	5.8
12	-1.7	-4.4	6.3

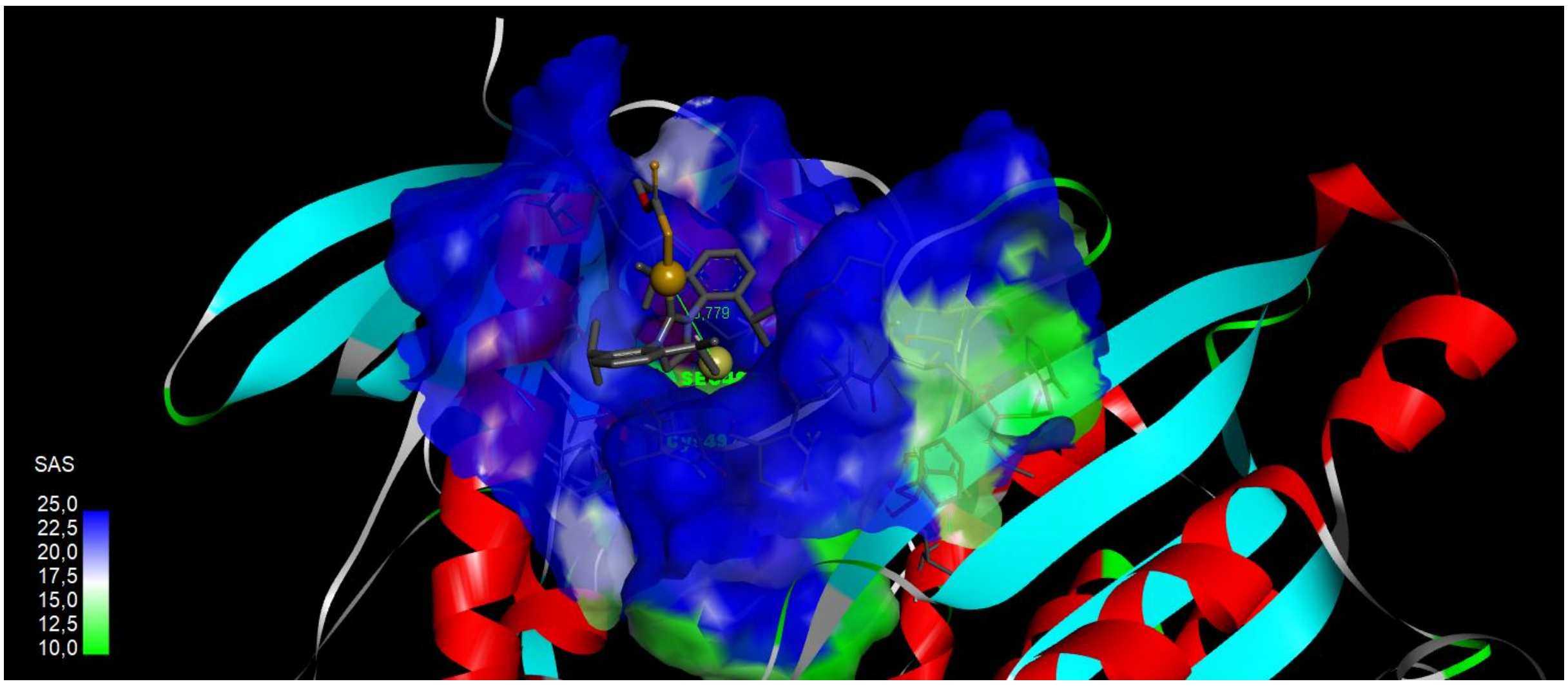

Figure S4. Overview of the top-scored binding pose for compound **1c.**

The enzyme is represented as a solvent-accessible surface around the active site (blue), ribbons depicting secondary structures, with SEC493 and its selenium atom (light yellow ball) represented explicitly. The inhibitor is shown as sticks with the Au atom depicted as a golden ball.

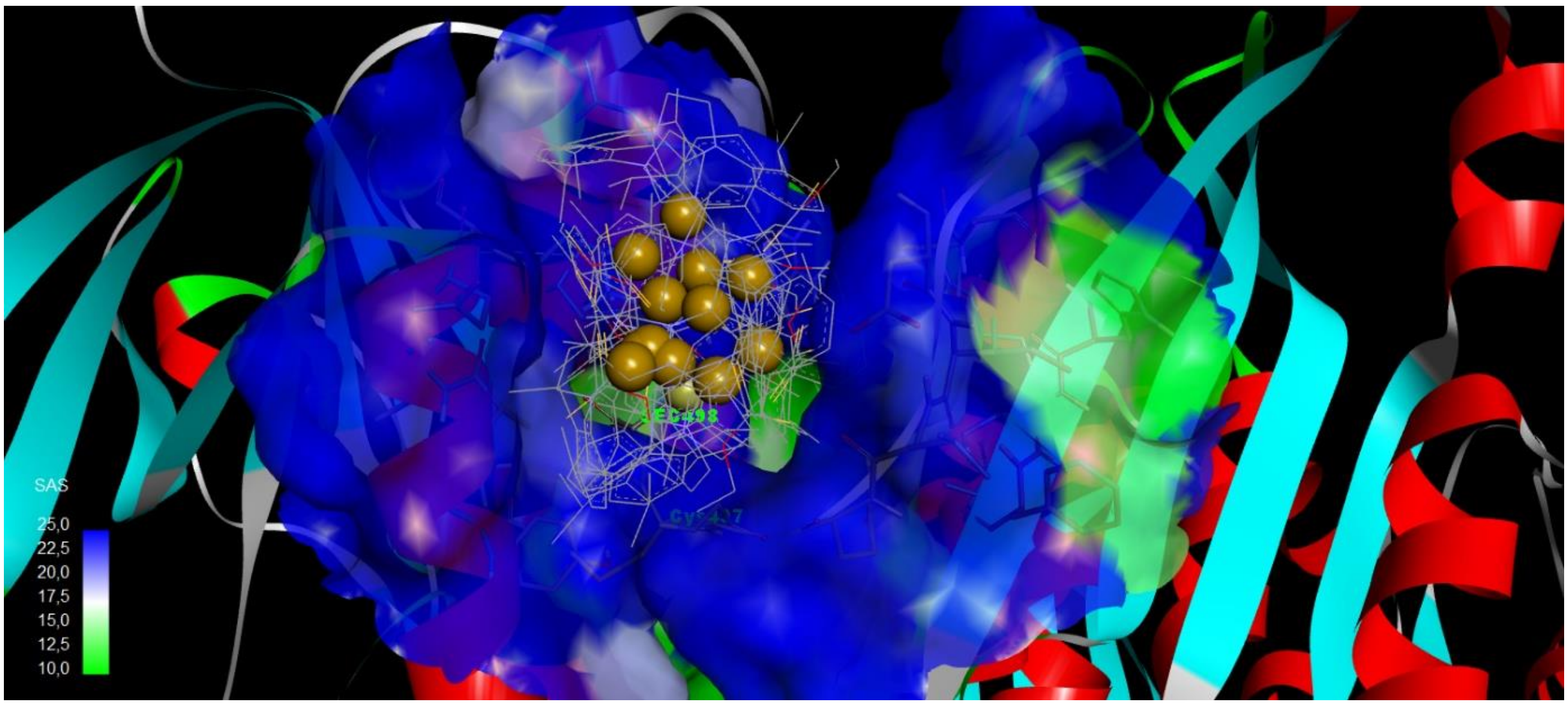

Figure S5. Population of all solutions found for **1c.**

The enzyme is represented as a solvent-accessible surface around the active site (blue), ribbons depicting secondary structures, with SEC493 and its selenium atom (light yellow ball) represented explicitly. The inhibitor is shown as thin lines with the Au atom depicted as a golden ball.

Table S4. Docking results for compound **1d.**

**Pose cluster #**	**Estimated Free Energy of Binding [kcal/mol]**	**Final Intermolecular Energy [kcal/mol]**	**Distance between Au and Se of SEC498 [Å]**
1	-3.6	-6.2	5.3
2	-3.3	-6.0	5.6
3	-3.3	-6.0	6.3
4	-3.3	-5.9	6.5
5	-2.9	-5.6	7.5
6	-2.4	-5.1	5.5
7	-2.3	-4.9	4.9
8	-2.1	-4.8	6.6
9	-2.0	-4.7	5.7
10	-1.9	-4.6	6.9
11	-1.8	-4.4	8.2
12	-1.7	-4.4	6.5
13	-1.7	-4.4	6.1
14	-1.6	-4.3	7.2

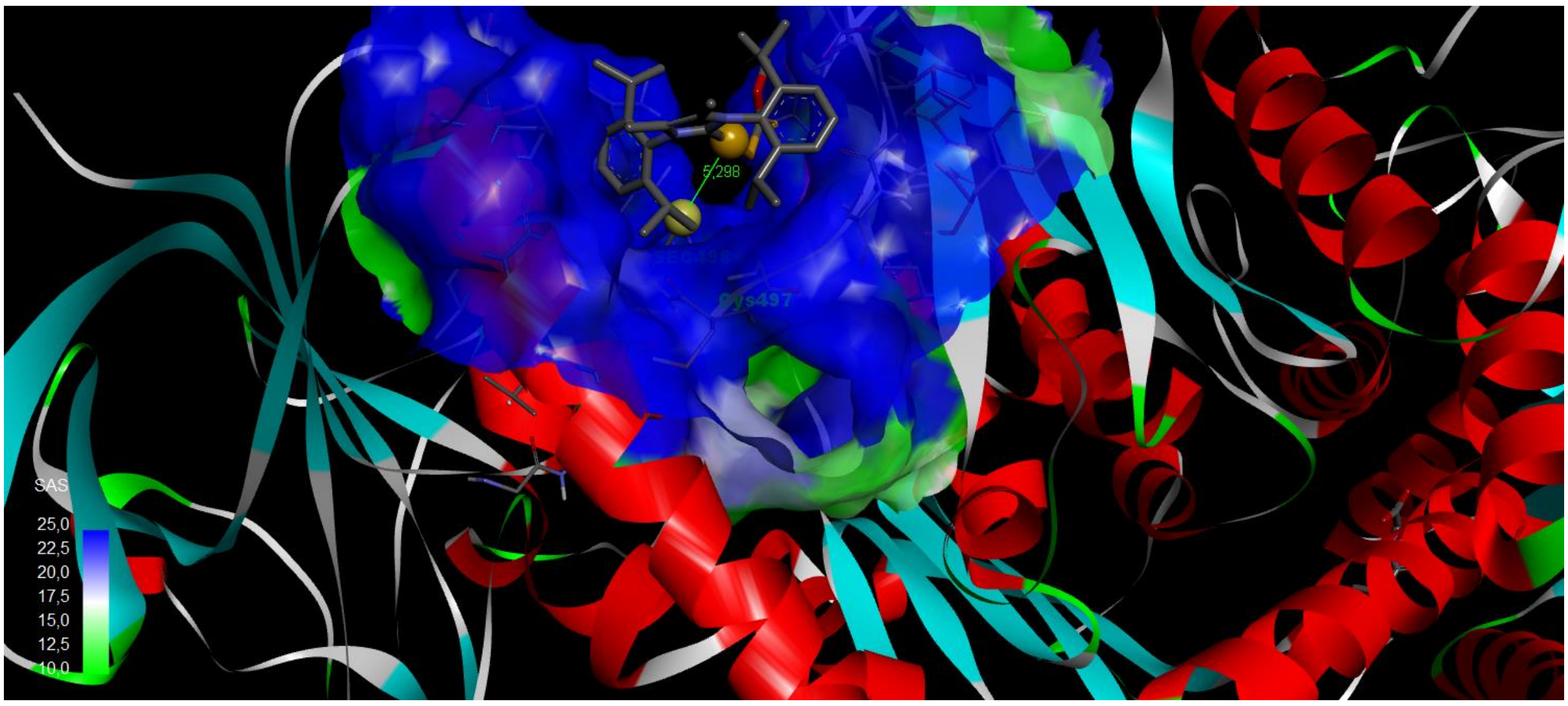

Figure S6. Overview of the top-scored binding pose for compound **1d.**

The enzyme is represented as a solvent-accessible surface around the active site (blue), ribbons depicting secondary structures, with SEC493 and its selenium atom (light yellow ball) represented explicitly. The inhibitor is shown as sticks with the Au atom depicted as a golden ball.

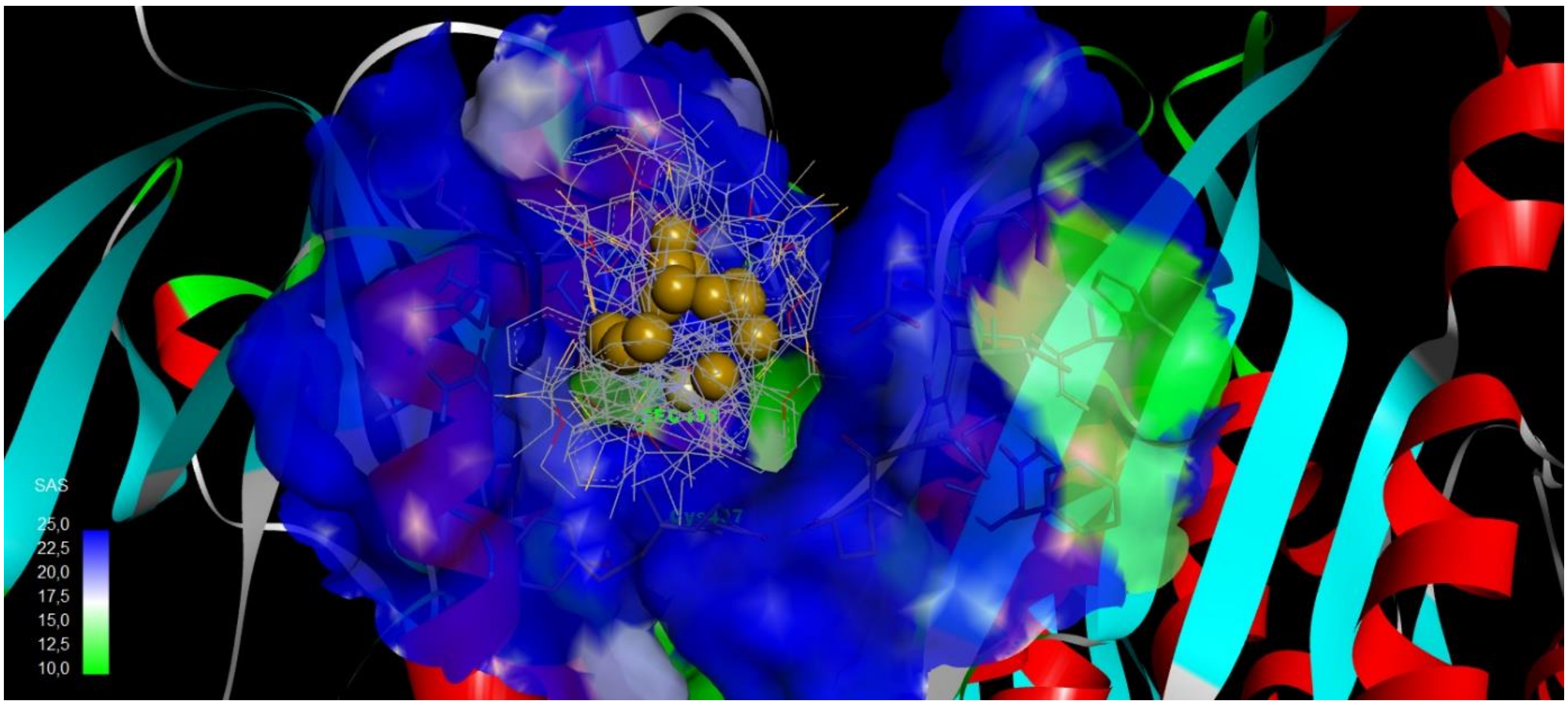

Figure S7. Population of all solutions found for **1d.**

The enzyme is represented as a solvent-accessible surface around the active site (blue), ribbons depicting secondary structures, with SEC493 and its selenium atom (light yellow ball) represented explicitly. The inhibitor is shown as thin lines with the Au atom depicted as a golden ball.

Table S5. Docking results for compound **1g.**

**Pose cluster #**	**Estimated Free Energy of Binding [kcal/mol]**	**Final Intermolecular Energy [kcal/mol]**	**Distance between Au and Se of SEC498 [Å]**
1	-5.6	-6.8	6.1
2	-5.5	-6.7	4.7
3	-5.5	-6.7	3.7
4	-5.2	-6.4	5.7
5	-5.2	-6.4	6.3
6	-5.1	-6.3	9.7
7	-4.9	-6.1	6.9
8	-4.7	-5.9	6.1
9	-4.6	-5.8	4.3
10	-4.5	-5.7	5.4
11	-4.2	-5.4	8.2
12	-4.0	-5.2	8.0
13	-3.3	-4.5	8.0

Table S6. Docking results for compound **1h.**

Pose cluster #	Estimated Free Energy of Binding [kcal/mol]	Final Intermolecular Energy [kcal/mol]	Distance between Au and Se of SEC498 [Å]
1	-9.0	-10.1	7.3
2	-7.2	-8.3	6.7
3	-6.0	-7.2	3.6
4	-4.9	-6.1	5.8
5	-4.8	-5.9	5.7
6	-4.0	-5.2	7.6
7	-3.9	-5.1	8.0
8	-3.8	-5.0	8.8
9	-3.7	-4.9	7.9
10	-3.4	-4.6	7.5
11	-3.4	-4.6	6.1
12	-3.4	-4.5	9.6
13	-3.3	-4.5	7.7
14	-2.8	-4.0	8.6
15	-2.5	-3.7	9.3
16	-2.2	-3.4	6.9

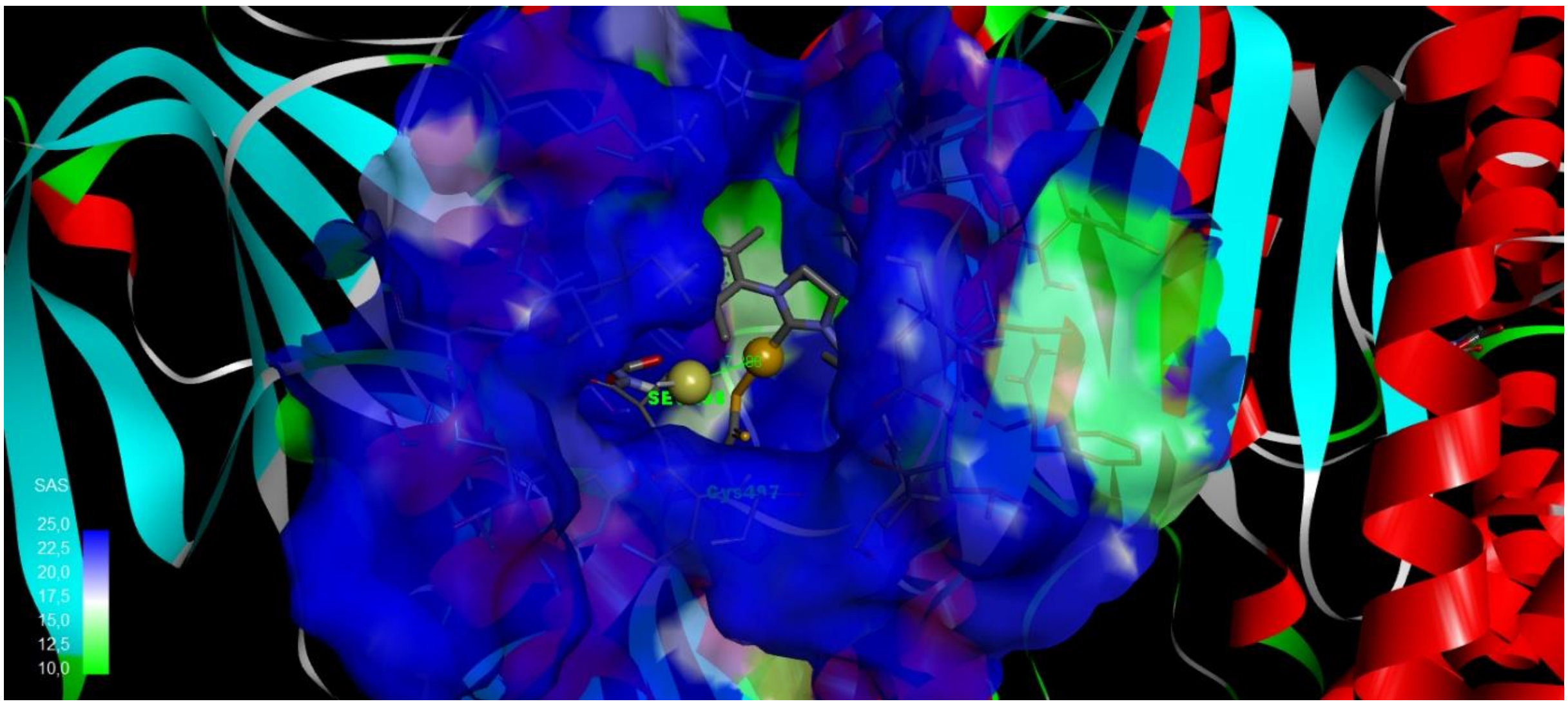

Figure S8. Overview of the top-scored binding pose for compound **1h.**

The enzyme is represented as a solvent-accessible surface around the active site (blue), ribbons depicting secondary structures, with SEC493 and its selenium atom (light yellow ball) represented explicitly. The inhibitor is shown as sticks with the Au atom depicted as a golden ball.

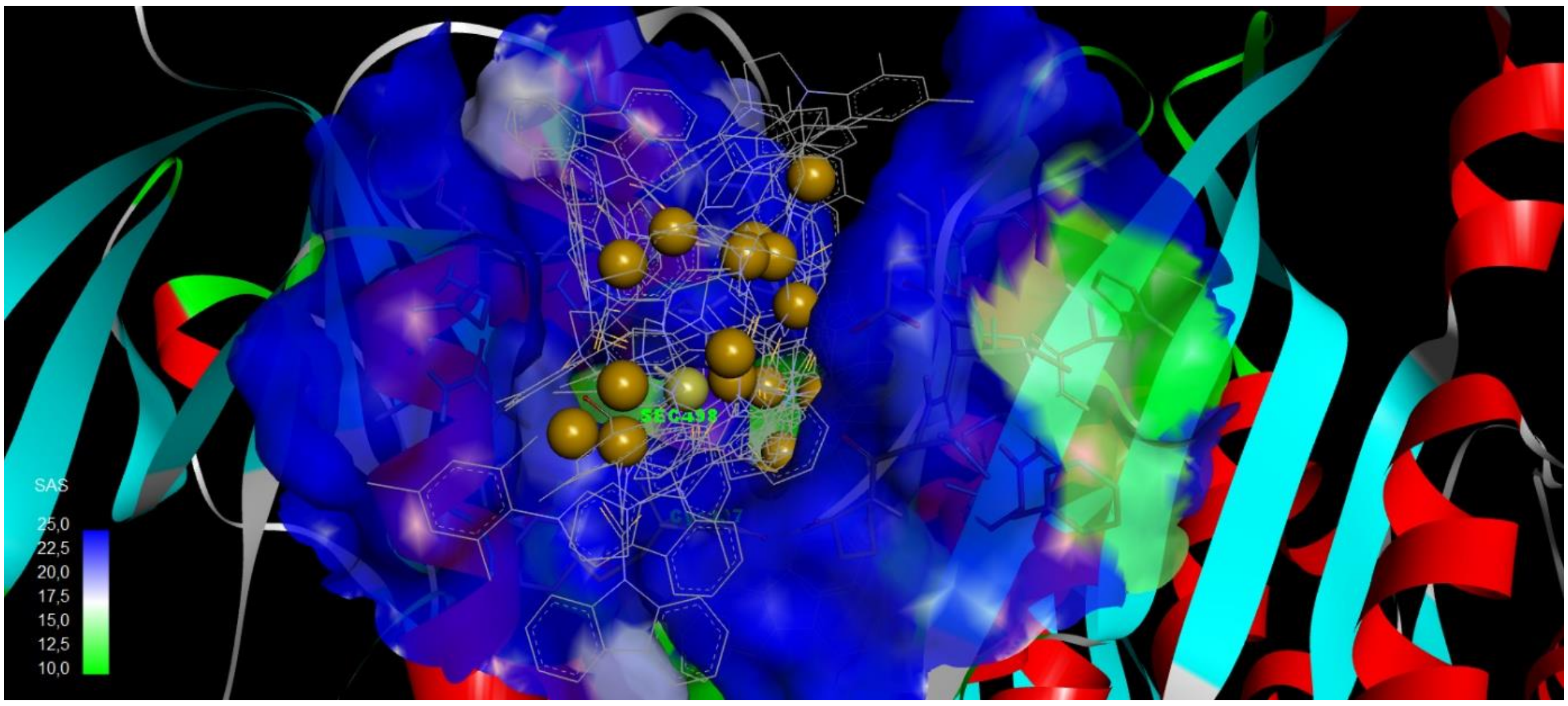

Figure S9. Population of all solutions found for **1h.**

The enzyme is represented as a solvent-accessible surface around the active site (blue), ribbons depicting secondary structures, with SEC493 and its selenium atom (light yellow ball) represented explicitly. The inhibitor is shown as thin lines with the Au atom depicted as a golden ball.

Table S7. Docking results for compound **1e.**

**Pose cluster #**	**Estimated Free Energy of Binding [kcal/mol]**	**Final Intermolecular Energy [kcal/mol]**	**Distance between Au and Se of SEC498 [Å]**
1	-5.1	-6.6	8.3
2	-4.3	-5.8	6.6
3	-4.2	-5.7	2.5
4	-3.8	-5.3	5.6
5	-3.8	-5.3	4.8
6	-3.7	-5.2	3.4
7	-3.7	-5.2	5.7
8	-3.7	-5.2	4.5
9	-3.7	-5.2	4.9
10	-3.6	-5.1	6.0
11	-3.4	-4.8	6.0
12	-3.3	-4.8	4.2
13	-3.1	-4.6	5.1
14	-3.0	-4.5	6.1

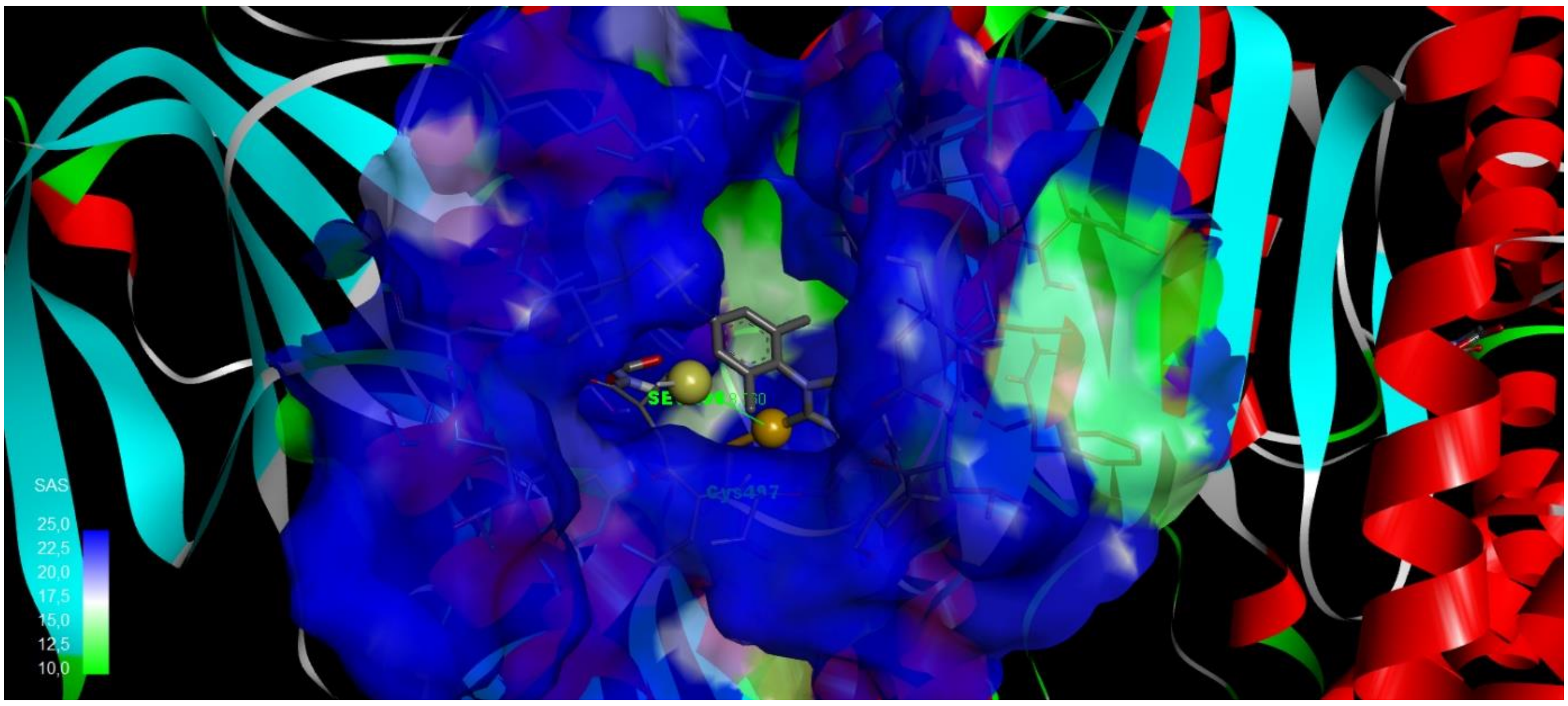

Figure S10. Overview of the top-scored binding pose for compound **1e.**

The enzyme is represented as a solvent-accessible surface around the active site (blue), ribbons depicting secondary structures, with SEC493 and its selenium atom (light yellow ball) represented explicitly. The inhibitor is shown as sticks with the Au atom depicted as a golden ball.

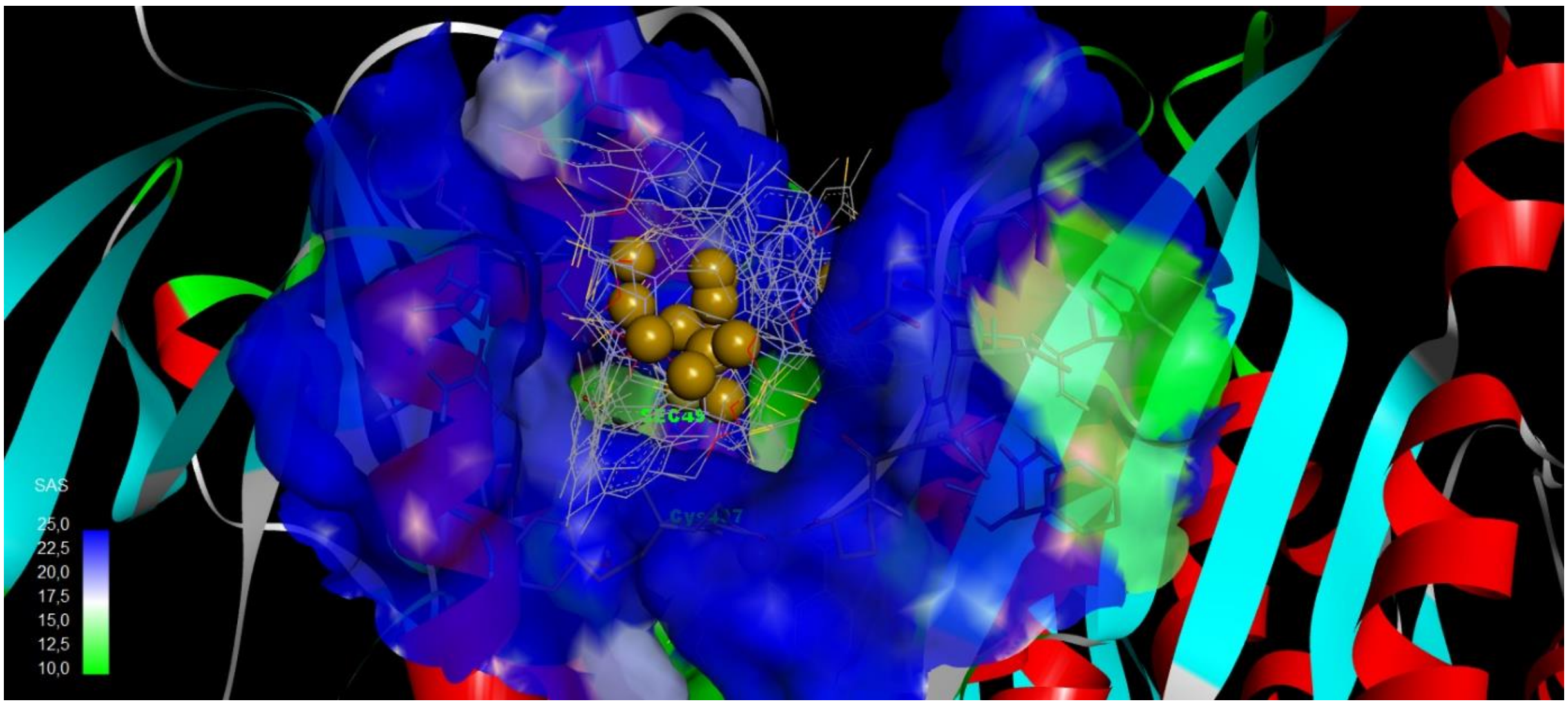

Figure S11. Population of all solutions found for **1e.**

The enzyme is represented as a solvent-accessible surface around the active site (blue), ribbons depicting secondary structures, with SEC493 and its selenium atom (light yellow ball) represented explicitly. The inhibitor is shown as thin lines with the Au atom depicted as a golden ball.

Table S8. Docking results for compound **1a.**

Pose cluster #	Estimated Free Energy of Binding [kcal/mol]	Final Intermolecular Energy [kcal/mol]	Distance between Au and Se of SEC498 [Å]
1	-3.3	-5.1	7.5
2	-3.2	-5.0	4.3
3	-3.2	-5.0	5.4
4	-2.8	-4.6	4.3
5	-2.6	-4.4	6.3
6	-4.9	-6.7	8.2
7	-4.8	-6.6	6.9
8	-4.6	-6.4	5.8
9	-4.6	-6.4	8.8
10	-4.5	-6.3	3.0
11	-4.1	-5.9	6.4
12	-3.8	-5.6	2.7
13	-3.7	-5.5	5.4
14	-3.6	-5.4	5.2

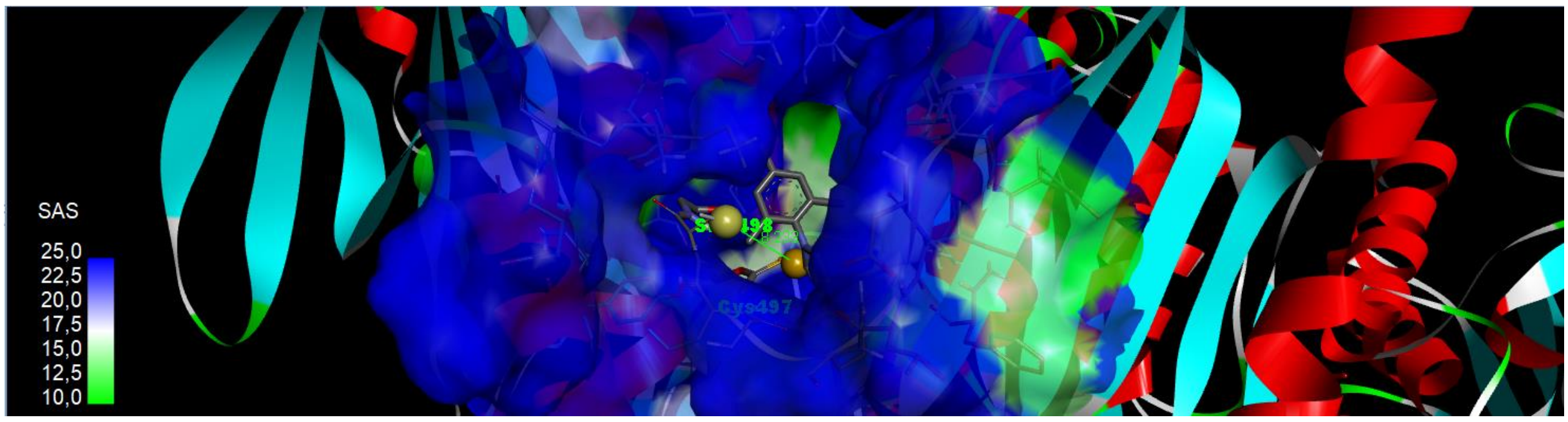

Figure S12. Overview of the top-scored binding pose for compound **1a.**

The enzyme is represented as a solvent-accessible surface around the active site (blue), ribbons depicting secondary structures, with SEC493 and its selenium atom (light yellow ball) represented explicitly. The inhibitor is shown as sticks with the Au atom depicted as a golden ball.

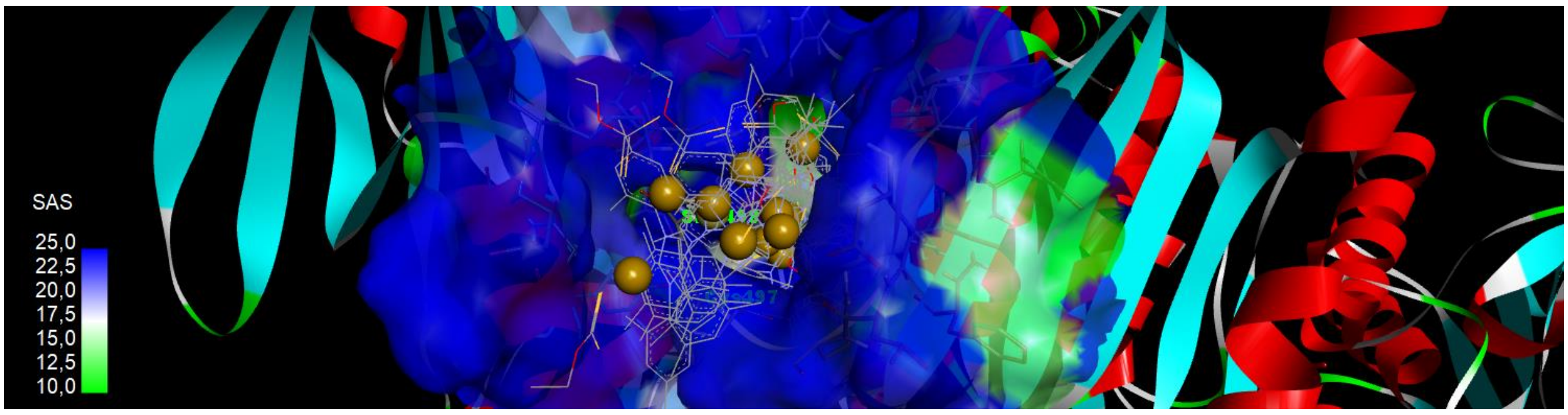

Figure S13. Population of all solutions found for **1a.**

The enzyme is represented as a solvent-accessible surface around the active site (blue), ribbons depicting secondary structures, with SEC493 and its selenium atom (light yellow ball) represented explicitly. The inhibitor is shown as thin lines with the Au atom depicted as a golden ball.

Table S9. Docking results for reference compound **auranofin.**

Pose cluster #	Estimated Free Energy of Binding [kcal/mol]	Final Intermolecular Energy [kcal/mol]	Distance between Au and Se of SEC498 [Å]
1	-3.3	-5.1	5.6
2	-3.3	-5.1	4.9
3	-3.1	-4.9	5.8
4	-3.1	-4.9	7.2
5	-3.0	-4.8	5.0
6	-2.8	-4.6	8.3
7	-2.8	-4.5	3.6
8	-2.3	-4.1	5.8
9	-2.2	-4.0	7.6
10	-2.1	-3.9	2.7
11	-4.7	-6.5	6.7
12	-4.3	-6.1	5.3
13	-4.0	-5.8	5.3
14	-3.9	-5.7	2.1

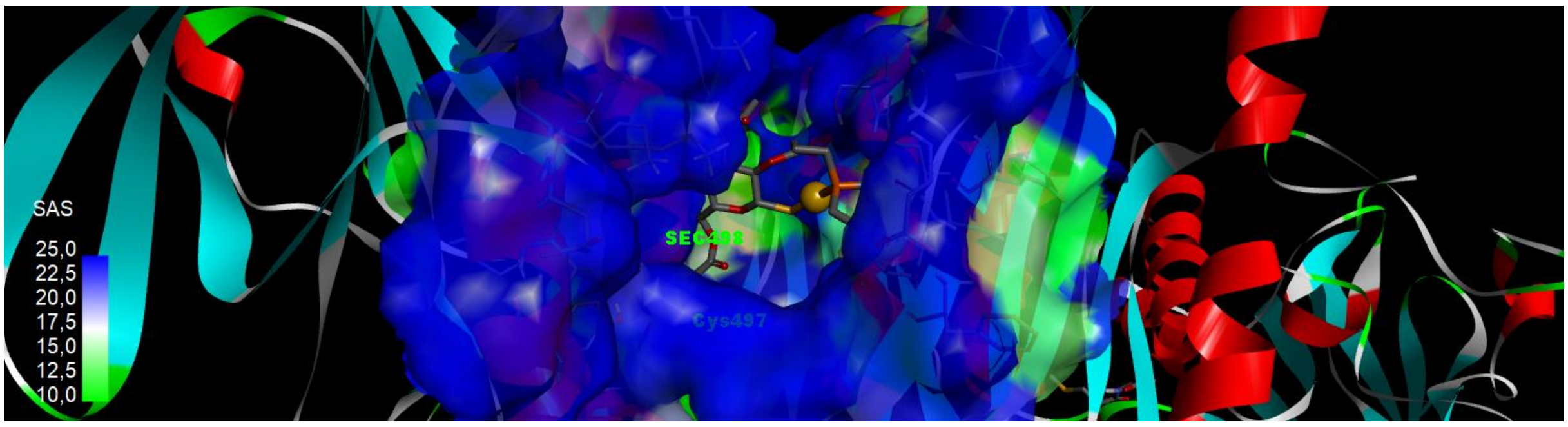

Figure S14. Overview of the top-scored binding pose for reference compound **auranofin.**

The enzyme is represented as a solvent-accessible surface around the active site (blue). ribbons depicting secondary structures. with SEC493 and its selenium atom (light yellow ball) represented explicitly. The inhibitor is shown as sticks with the Au atom depicted as a golden ball.

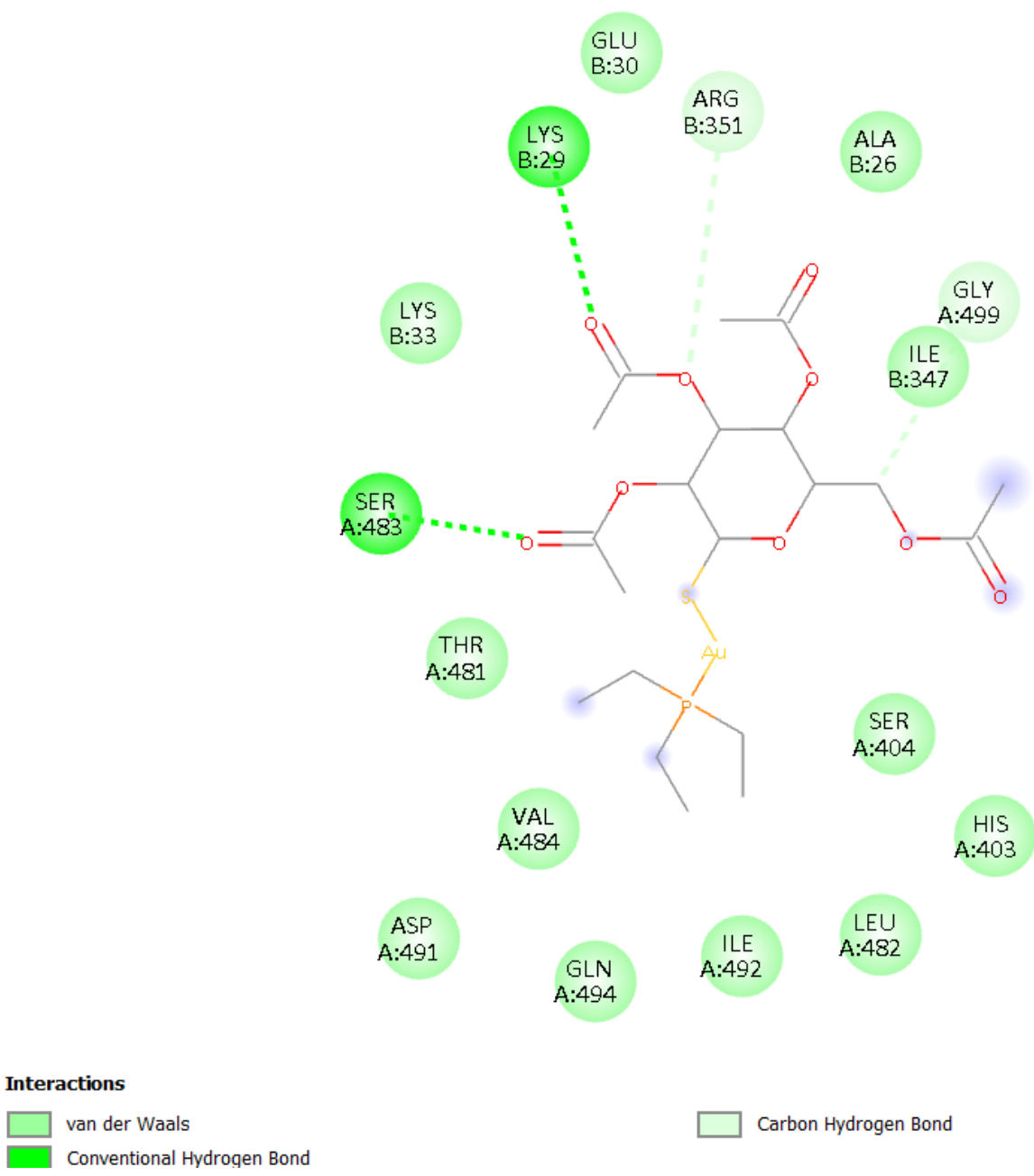

Figure S15. Interaction diagram for the top-scored binding pose for reference compound **auranofin**.

For **auranofin**, the predicted top scored binding mode places the acetylated thioglucose moiety within the intersubunit region, while the Au-containing phosphine–thiolate fragment extends toward the adjacent cleft. The ligand is stabilized by conventional hydrogen-bond interactions with Lys29 of subunit B and Ser483 of subunit A. Additional contacts involve residues from both subunits, including Ala26, Glu30, Lys33, Arg351, Ser404, His403, Thr481, Val484, Ile492, Leu482, and Gly499, indicating that in this pose, auranofin occupies a relatively extended interfacial pocket. The gold atom in this binding pose is located at a distance of 6.7 Å from Se of the selenocysteine SEC498.

In comparison, complex **1f** , in the top pose, adopts a more focused orientation toward the catalytic redox center (see main text and Figure 5 for details).

Overall , in the whole population of the solutions found for **auranofin**, the average Au ⋯ Se SEC498 distance is 5.42 Å ± 1.75 (as compared to 5.03 Å ± 1.28 found for **1f**). This seems fitting to the weak correlation described in the main text (see Figure 6). It is though to be noted that the limitations mentioned in the main text apply similarly to the case of auranofin.

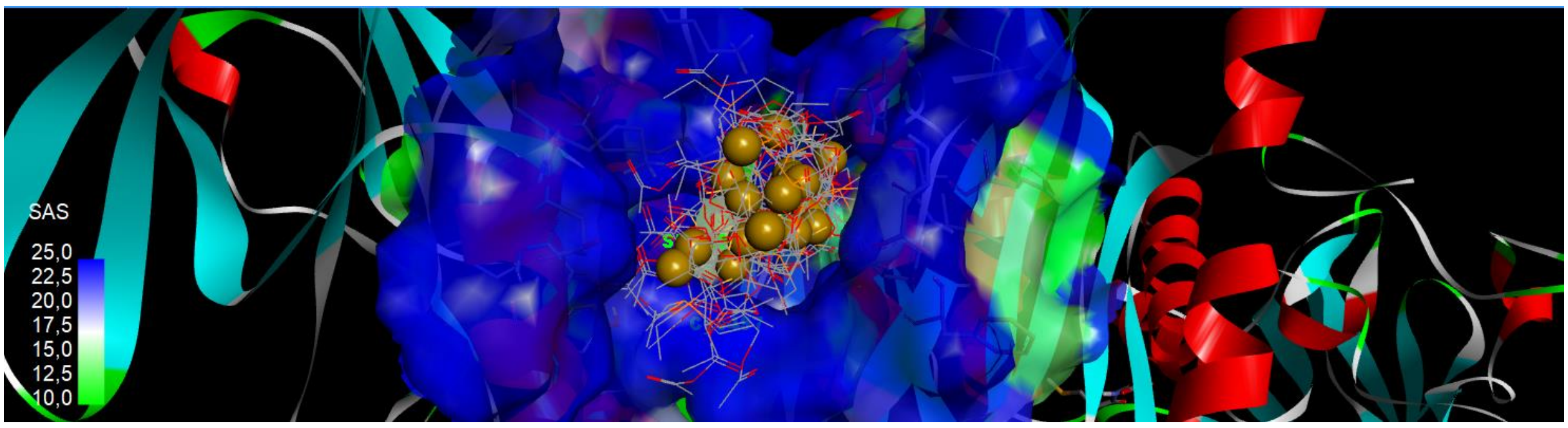

Figure S16. Population of all solutions found for reference compound **auranofin.**

The enzyme is represented as a solvent-accessible surface around the active site (blue). ribbons depicting secondary structures. with SEC493 and its selenium atom (light yellow ball) represented explicitly. The inhibitor is shown as thin lines with the Au atom depicted as a golden ball.

4. Copies of spectra

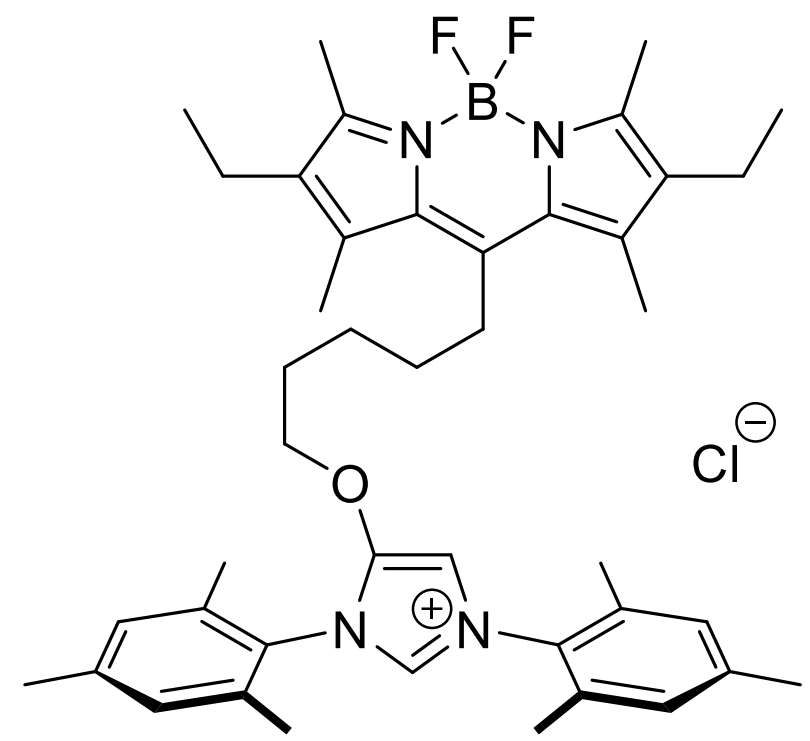

4

^{1}H NMR (400 MHz, $CDCl_3$)

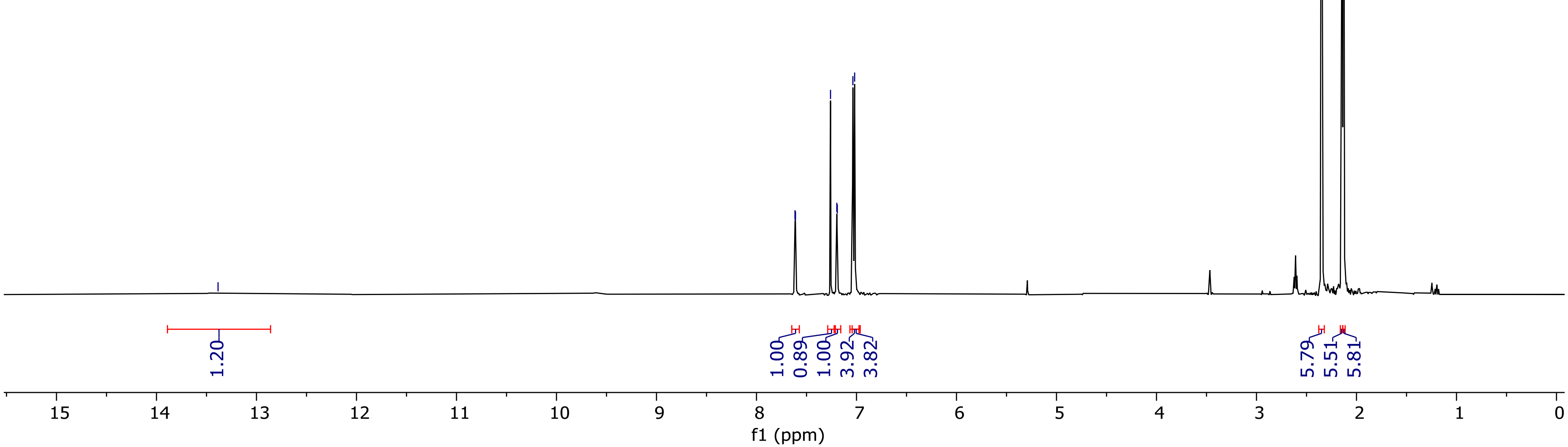

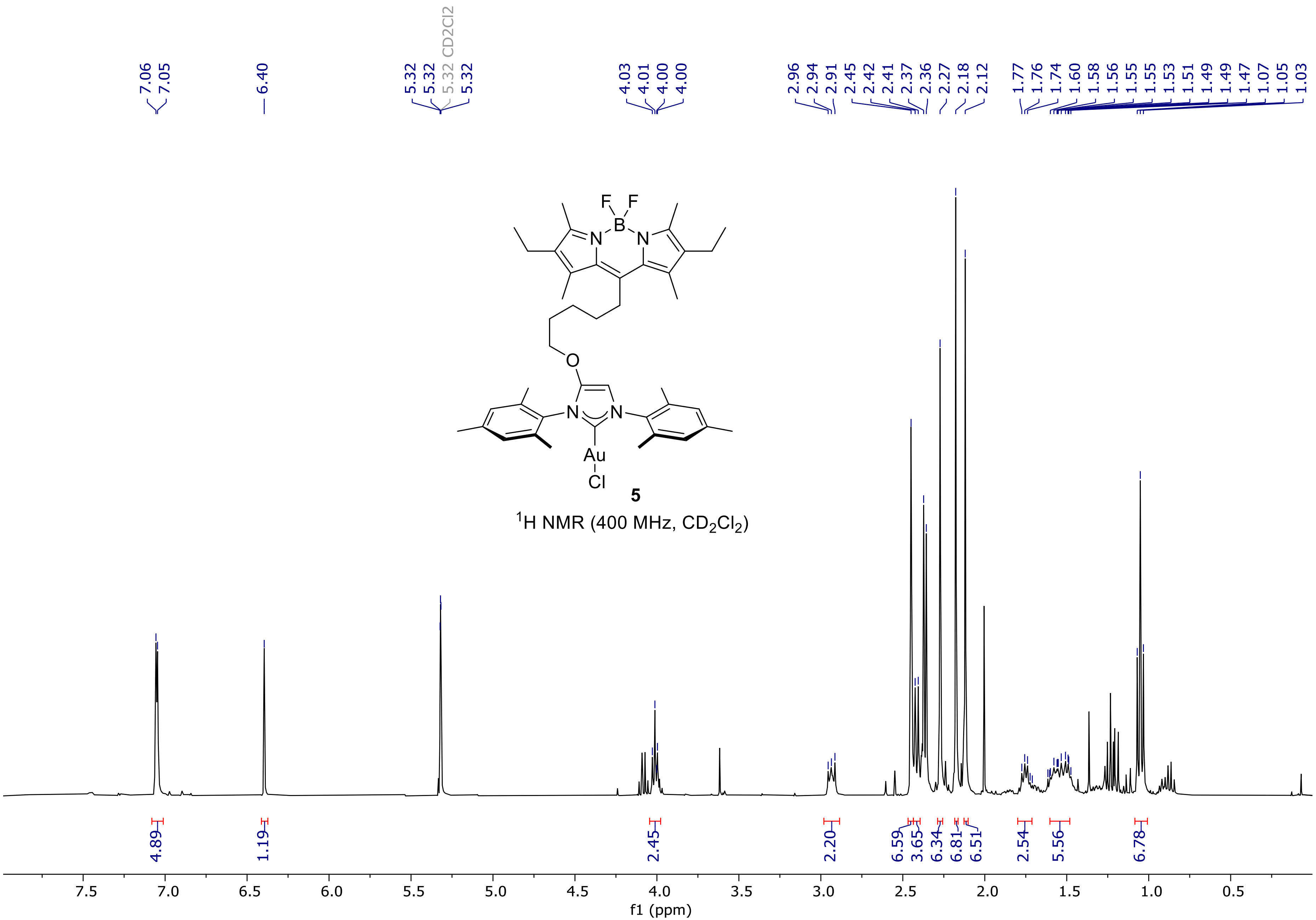
7.06
7.05
6.40
5.32
5.32
5.32 CD2Cl2
5.32
4.03
4.01
4.00
4.00
2.96
2.94
2.91
2.45
2.42
2.41
2.37
2.36
2.27
2.18
2.12
1.77
1.76
1.74
1.60
1.58
1.56
1.55
1.55
1.53
1.51
1.49
1.49
1.47
1.07
1.05
1.03
F
F
B
N
N
O
N
N
Au
Cl
5
^{1}H NMR (400 MHz, CD_2Cl_2)
4.89
1.19
2.45
2.20
6.59
3.65
6.34
6.81
6.51
2.54
5.56
6.78
7.5
7.0
6.5
6.0
5.5
5.0
4.5
4.0
3.5
3.0
2.5
2.0
1.5
1.0
0.5
f1 (ppm)

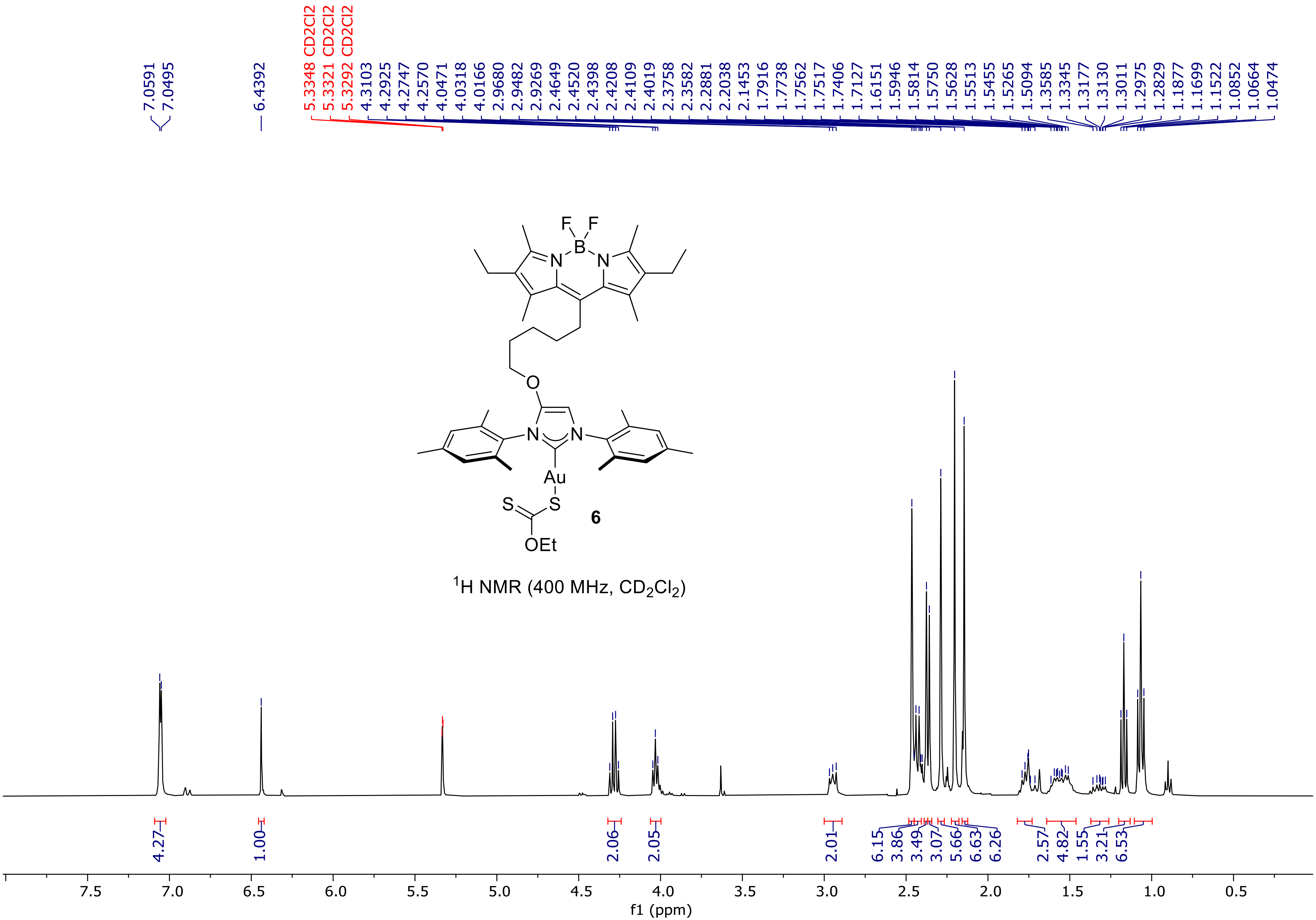
7.0591
7.0495
6.4392
5.3348 CD2Cl2
5.3321 CD2Cl2
5.3292 CD2Cl2
4.3103
4.2925
4.2747
4.2570
4.0471
4.0318
4.0166
2.9680
2.9482
2.9269
2.4649
2.4520
2.4398
2.4208
2.4109
2.4019
2.3758
2.3582
2.2881
2.2038
2.1453
1.7916
1.7738
1.7562
1.7517
1.7406
1.7127
1.6151
1.5946
1.5814
1.5750
1.5628
1.5513
1.5455
1.5265
1.5094
1.3585
1.3345
1.3177
1.3130
1.3011
1.2975
1.2829
1.1877
1.1699
1.1522
1.0852
1.0664
1.0474
F
F
B
N
N
O
N
N
Au
S
S
OEt
6
^{1}H NMR (400 MHz, CD_2Cl_2)
4.27
1.00
2.06
2.05
2.01
6.15
3.86
3.49
3.07
5.66
6.63
6.26
2.57
4.82
1.55
3.21
6.53
7.5
7.0
6.5
6.0
5.5
5.0
4.5
4.0
3.5
3.0
2.5
2.0
1.5
1.0
0.5
f1 (ppm)

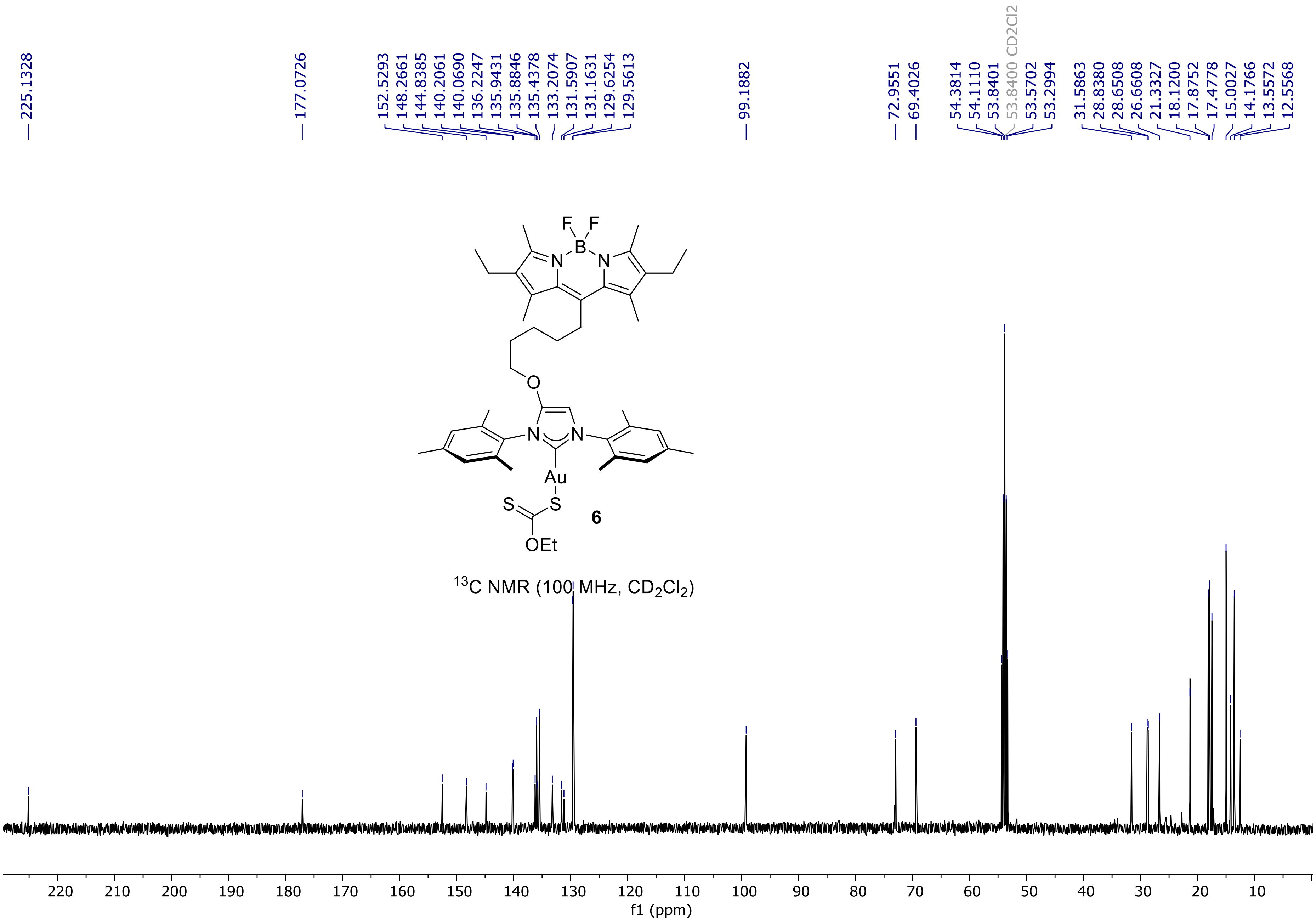
225.1328
177.0726
152.5293
148.2661
144.8385
140.2061
140.0690
136.2247
135.9431
135.8846
135.4378
133.2074
131.5907
131.1631
129.6254
129.5613
99.1882
72.9551
69.4026
54.3814
54.1110
53.8401
53.8400 CD2Cl2
53.5702
53.2994
31.5863
28.8380
28.6508
26.6608
21.3327
18.1200
17.8752
17.4778
15.0027
14.1766
13.5572
12.5568
F
F
B
N
N
O
N
N
Au
S
S
OEt
6
^{13}C NMR (100 MHz, CD_2Cl_2)
220
210
200
190
180
170
160
150
140
130
120
110
100
90
80
70
60
50
40
30
20
10
f1 (ppm)

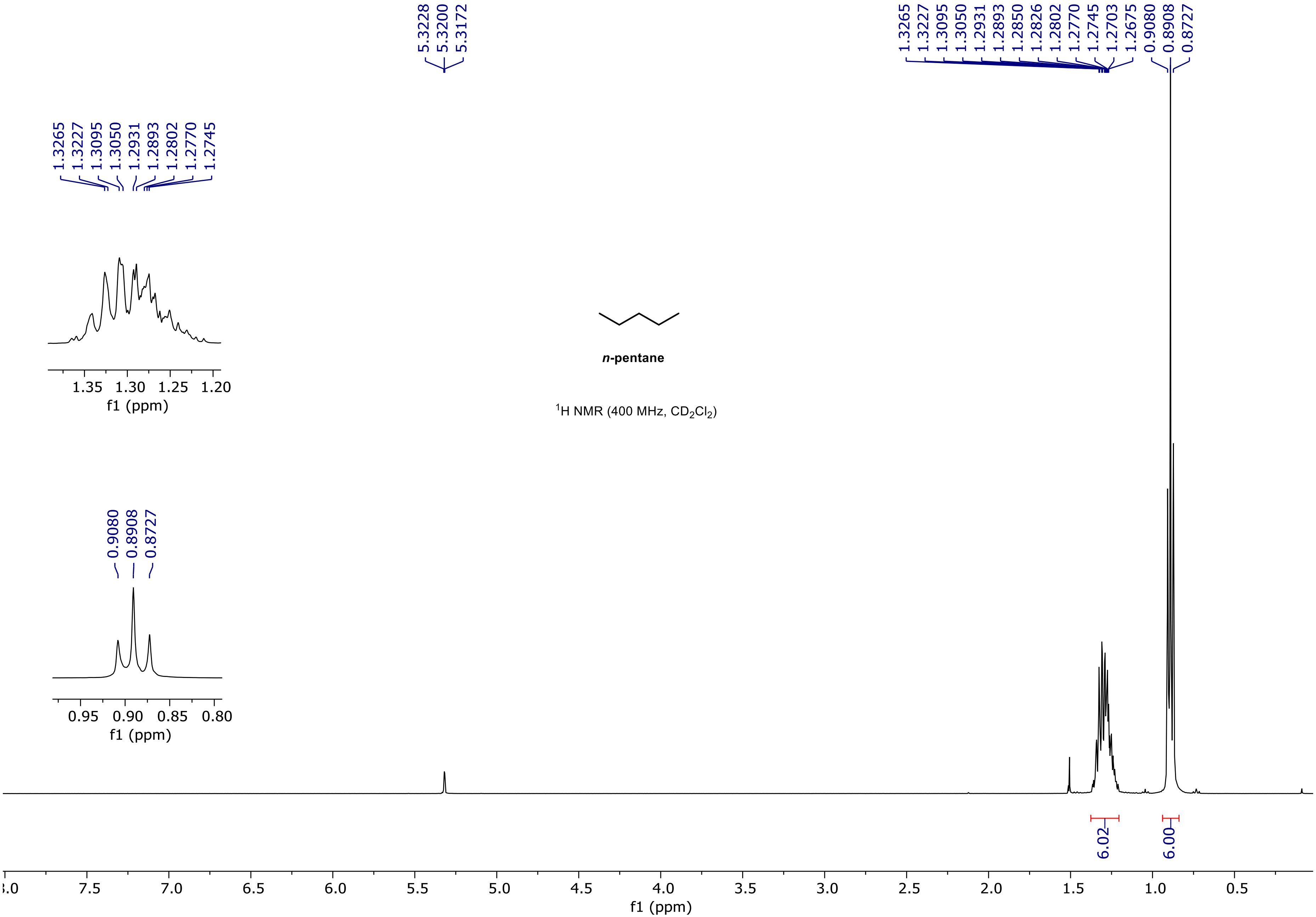

5.3228
5.3200
5.3172
1.3265
1.3227
1.3095
1.3050
1.2931
1.2893
1.2850
1.2826
1.2802
1.2770
1.2745
1.2703
1.2675
0.9080
0.8908
0.8727
n-pentane
^{1}H NMR (400 MHz, CD_2Cl_2)
6.02
6.00
f1 (ppm)

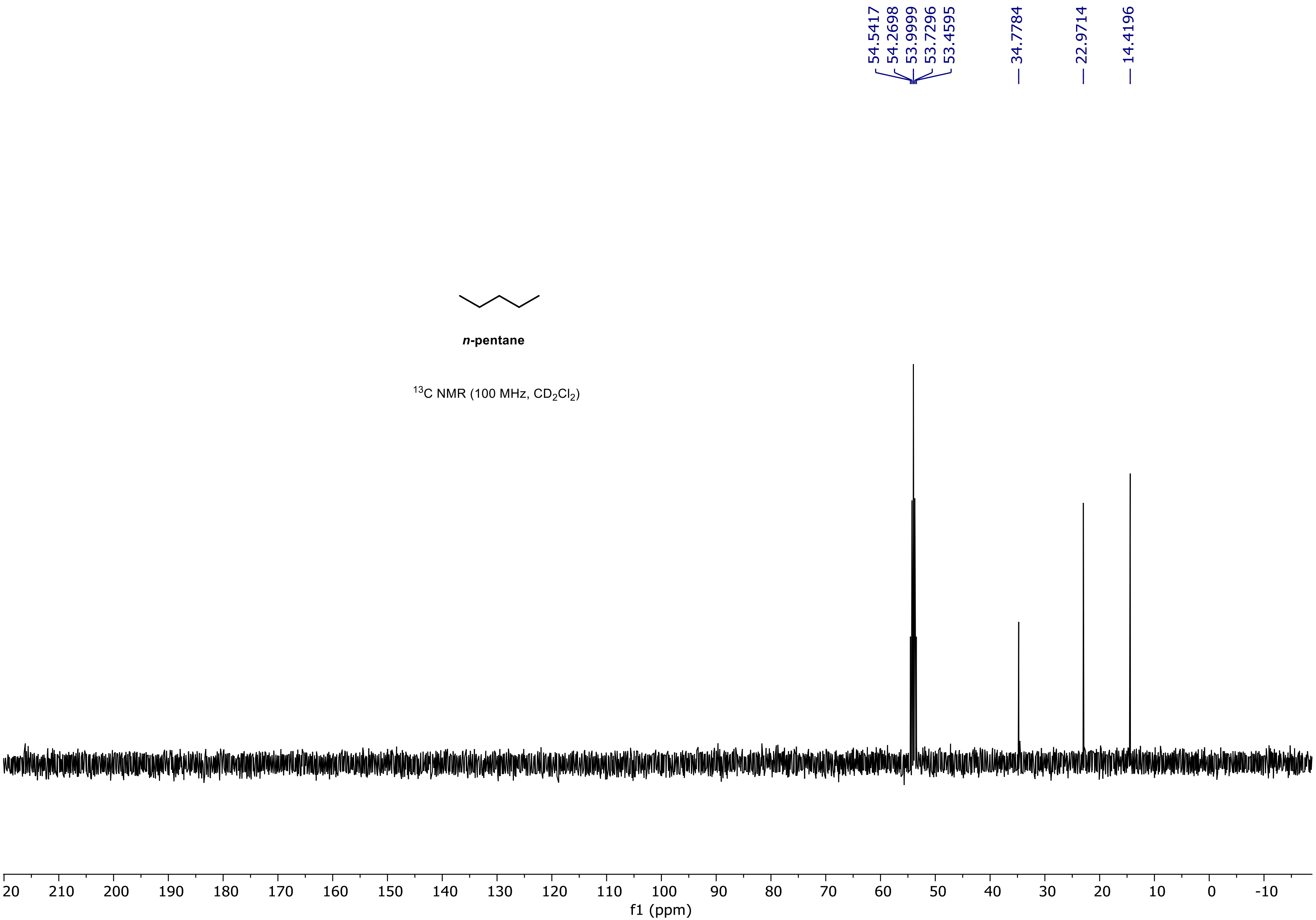

54.5417
54.2698
53.9999
53.7296
53.4595
34.7784
22.9714
14.4196
n-pentane
^{13}C NMR (100 MHz, CD_2Cl_2)
20 210 200 190 180 170 160 150 140 130 120 110 100 90 80 70 60 50 40 30 20 10 0 -10
f1 (ppm)

5. References

1. M. Michalak, P. Czerwiński, K. Śniady-Maciążek, S. Musioł, O. Danylyuk, M. Wierzbicki, M. Tomasini and A. Poater, Chiral NHC Ligands for Enantioselective Gold(I) Catalysis Under Aerobic Conditions: the Importance of Conformational Flexibility and Steric Hindrance of NHC Ligand on Reactivity *Chem. Eur. J.*, 2024, **30**, e202303241.
2. S. Chakraborty, A. Gorski, O. Danylyuk, K. Niemirowicz-Laskowska, H. Car and M. Michalak, NHC–Au–xanthate complexes *Chem. Commun.*, 2025, **61**, 1697-1700. DOI: 10.1039/D4CC05193A
3. Y. Shinozaki, S. Popov and H. Plenio, Fluorescent organometallic dyads and triads: establishing spatial relationships *Chem. Sci.*, 2023, **14**, 350-361. DOI: 10.1039/D2SC04869H
4. L. Benhamou, N. Vujkovic, V. César, H. Gornitzka, N. Lugan and G. Lavigne, Facile Derivatization of a "Chemo-active" NHC Incorporating an Enolate Backbone and Relevant Tuning of Its Electronic Properties *Organometallics*, 2010, **29**, 2616-2630. DOI: 10.1021/om1003607
5. O. Halter, I. Fernández and H. Plenio, Fluorescence resonance energy transfer (FRET) for the verification of dual gold catalysis *Chem. Eur. J.*, 2017, **23**, 711-719. DOI: 10.1039/C7CC07018G
